# Endothelial *Rictor* is crucial for midgestational development and sustained and extensive FGF2-induced neovascularization in the adult

**DOI:** 10.1038/srep17705

**Published:** 2015-12-04

**Authors:** Fabio Aimi, Stavroula Georgiopoulou, Ina Kalus, Fabienne Lehner, Alica Hegglin, Përparim Limani, Vinicius Gomes de Lima, Markus A. Rüegg, Michael N. Hall, Nicole Lindenblatt, Elvira Haas, Edouard J. Battegay, Rok Humar

**Affiliations:** 1 Department of Internal Medicine, University Hospital, CH-8091 Zürich, Switzerland; 2 Division of Plastic and Reconstructive Surgery, University Hospital, CH-8091 Zürich, Switzerland; 3 Biozentrum, University of Basel, CH-4057 Basel, Switzerland; 4 Division of Visceral and Transplant Surgery, University Hospital, CH-8091 Zürich, Switzerland; 5 Zürich Center for Integrative Human Physiology, University of Zürich, Switzerland; 6 Center of Competence Multimorbidity and University Research Priority Program “Dynamics of Healthy Aging”, University of Zurich, Switzerland

## Abstract

To explore the general requirement of endothelial mTORC2 during embryonic and adolescent development, we knocked out the essential mTORC2 component *Rictor* in the mouse endothelium in the embryo, during adolescence and in endothelial cells *in vitro*. During embryonic development, *Rictor* knockout resulted in growth retardation and lethality around embryonic day 12. We detected reduced peripheral vascularization and delayed ossification of developing fingers, toes and vertebrae during this confined midgestational period. *Rictor* knockout did not affect viability, weight gain, and vascular development during further adolescence. However during this period, *Rictor* knockout prevented skin capillaries to gain larger and heterogeneously sized diameters and remodeling into tortuous vessels in response to FGF2. *Rictor* knockout strongly reduced extensive FGF2-induced neovascularization and prevented hemorrhage in FGF2-loaded matrigel plugs. *Rictor* knockout also disabled the formation of capillary-like networks by FGF2-stimulated mouse aortic endothelial cells *in vitro*. Low RICTOR expression was detected in quiescent, confluent mouse aortic endothelial cells, whereas high doses of FGF2 induced high RICTOR expression that was associated with strong mTORC2-specific protein kinase Cα and AKT phosphorylation. We demonstrate that the endothelial FGF-RICTOR axis is not required during endothelial quiescence, but crucial for midgestational development and sustained and extensive neovascularization in the adult.

Controlled angiogenesis is required during early development to generate the peripheral vasculature and in the adult to regenerate damaged tissue and the endometrial lining. In contrast, uncontrolled angiogenesis, which is characterized by an unorganized, tortuous and hyper-permeable vascular network, is induced in many pathological processes, including tumor growth, metastasis, diabetic retinopathy, and arthritis^1^,^2^. Angiogenic cascades are initiated by potent angiogenic molecules, such as fibroblast growth factor (FGF) or vascular endothelial growth factor (VEGF). FGF and VEGF both activate the protein kinase mammalian target of rapamycin (mTOR)^3^,^4^ via phosphatidylinositide 3-kinase (PI3K) activation. Pharmacological inhibition of mTOR-linked signaling has been shown to reduce tumor angiogenesis and tumor growth in various experimental models. The mTOR inhibitor, rapamycin, suppresses tumor angiogenesis *in vivo* by downregulating VEGF^5^,^6^. Furthermore, dual PI3K and mTOR inhibitors block VEGF-induced neovascularization in mice^7^,^8^,^9^. Similarly, dual mTOR inhibitors have been shown to considerably reduce angiogenesis and regrowth compared to rapamycin alone^10^.

Mammalian TOR acts as the core protein kinase in two different multi-protein complexes: mTOR complex (mTORC) 1 and 2. In most tissues, rapamycin is largely selective for mTORC1^11^.

The complex of interest in this study is mTORC2, which contains the essential regulatory proteins RICTOR, mSIN1, and mLST8. mTORC2 integrates signals from growth factors to regulate cell survival or cytoskeleton organization. In addition, mTORC2 phosphorylates AGC kinase family members, such as AKT and protein kinase Cα (PKCα)^11^ and is implicated in the epithelial-mesenchymal transition (EMT)^12^,^13^. Embryos lacking *Rictor* or *Mlst8* in the whole body are growth retarded and die at around midgestation^14^,^15^. We have previously shown that hypoxia, a main stimulus for angiogenesis, induces transient mTORC1 activity, whereas mTORC2-induced AKT activation is sustained and critical for endothelial proliferation^16^. This suggested a specific function of mTORC2 in angiogenesis *in vivo*. Recently, distinct mTORC2 signaling pathways have been reported to regulate endothelial cell proliferation and vascular assembly in response to VEGF^17^.

High doses of rapamycin or its prolonged delivery can also block mTORC2 in endothelial cells^18^. Thus, rapamycin-based inhibitors cannot differentiate between the functions of mTORC1 and mTORC2 and specific mTORC2 inhibitors are not available. Here we therefore deleted *Rictor* in the endothelium to study the general requirement of endothelial mTORC2 during embryonic and adolescent development. Our second main aim was to elucidate whether endothelial RICTOR participates in vascular changes upon wounding and extensive angiogenic stimulation in the existing capillary bed and during *de novo* angiogenesis.

## Results

### Loss of endothelial homozygous *Rictor* results in embryonic lethality around embryonic day (E) 11.5–12.5

**Figure 1:**
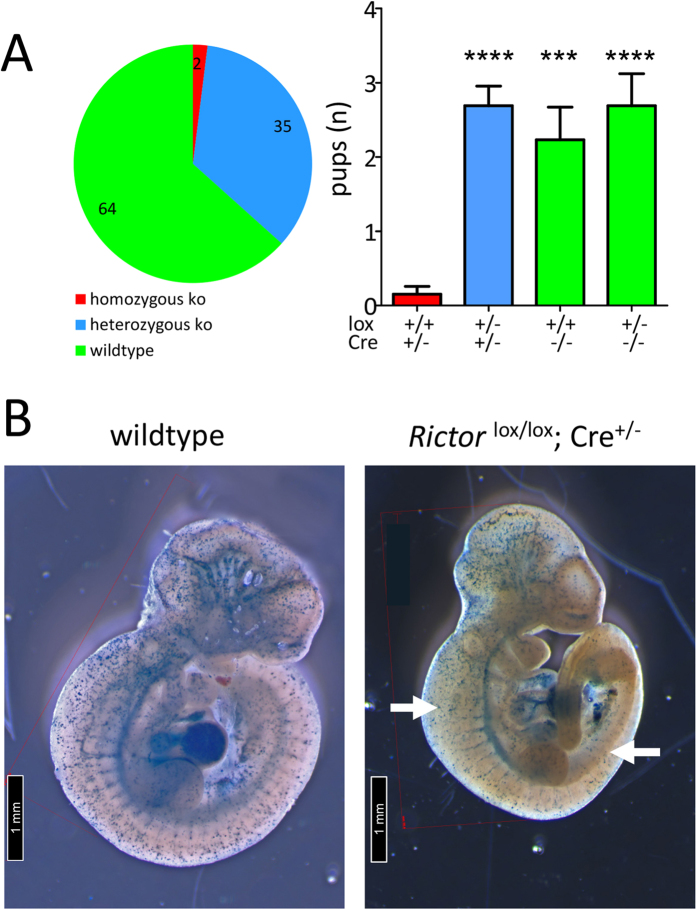
Constitutive homozygous endothelial *Rictor* knockout during embryonic development is generally lethal. (**A**) *Rictor*^flox/flox^ females were mated with *Rictor*^flox/−^; VE-Cadherin-Cre^+/−^; LacZ reporter^+/+^ males to generate homozygous deletion of *Rictor* in the endothelium. Litter genotypes were determined by qPCR and are displayed as total distribution and average number of pups per genotype (n_total pups_ = 101, ****P < 0.0001, ***P < 0.001, 1-way ANOVA with Bonferroni multiple comparison) (**B)**. The abovementioned breeding scheme was used to isolate embryonic day (E) 10.5 wildtype and endothelial *Rictor* knockout embryos. Representative β-galactosidase staining (blue) of E10.5 embryos shows the active sites of VE-Cadherin-Cre recombination. Arrows on the right indicate reduced peripheral LacZ staining in *Rictor* knockout embryos (n = 7 of 11).

**Figure 2:**
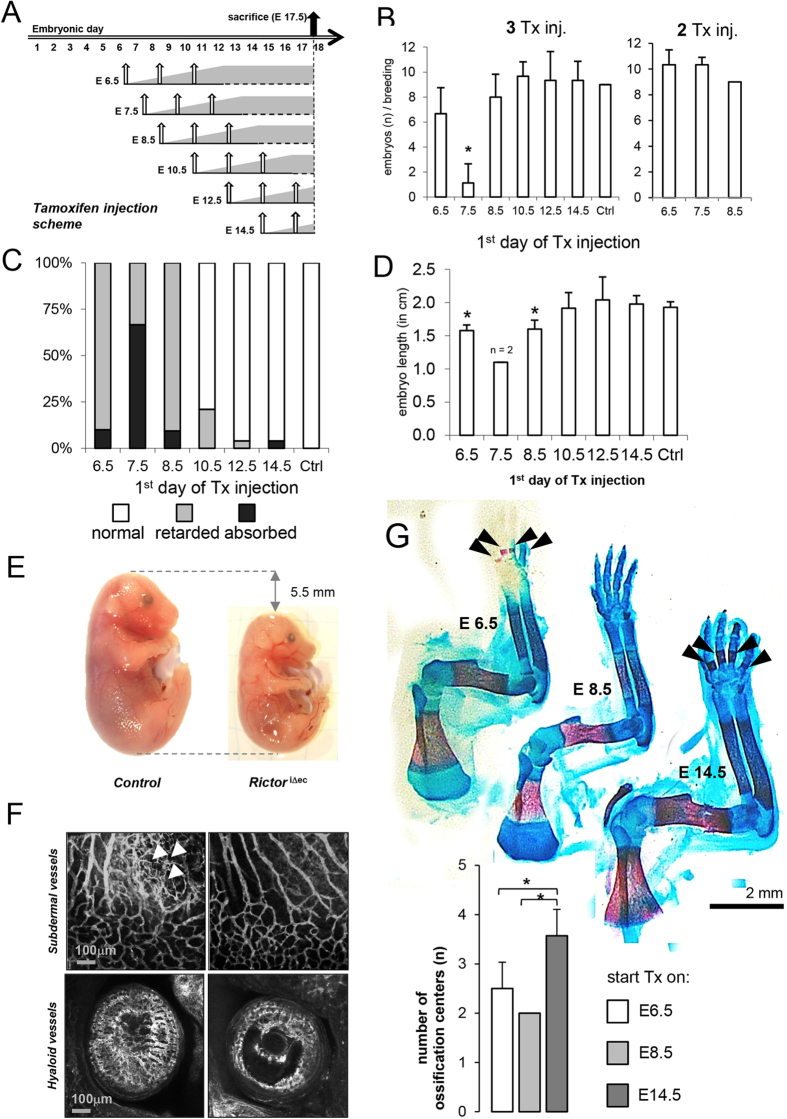
Lethality and growth retardation of induced endothelial *Rictor* knockout mice peaks around E12. **(A)** Tamoxifen (Tx) injection scheme: Three doses of Tx were administered to pregnant females every 48 hours, beginning on E6.5, E7.5, E8.5, E10.5, E12.5, and E14.5. Pregnant females were sacrificed, and embryos were harvested on E17.5 for further histological analysis. Grey area symbolically depicts stepwise *Rictor* knockout. Control females were injected with corn oil. (**B)** *Rictor*^iΔec^ mice exhibit decreased litter size. Statistical analysis of the litter size of individual breedings (n_Eday_) after three and two Tx injections at different time points (n_E6.5_ = 3, n_E7.5_ = 3, n_E8.5_ = 4, n_E10.5_ = 3, n_E12.4_ = 4, n_E14.5_ = 4,) compared to controls (n = 7). *P < 0.05, Student’s t-test. **(C)** Statistical analysis of normal, growth retarded, and absorbed embryos per breeding after Tx-injection at different time points. Total litter size for breeding at each time point was set to 100%, n_breedings_ = 3. (**D)** Statistical analysis of the length of surviving embryos per litter at different starting time points of Tx injections. n = 4 (embryos per time point, length was measured in both extremities), *P < 0.01 compared to controls. Student’s t-test. (**E)** Representative picture of a E17.5 *Rictor*^iΔec^ embryo that was induced by Tx on E8.5 in comparison to a wildtype embryo. (**F**) *Rictor*^iΔec^ embryos displayed distinct vascular deficits in the eye when Tx injections started on E7.5 and embryos harvested at E12.5 and stained with the vessel-specific antibody, endomucin. Scale bar = 100 μm. Arrows indicate angiogenic sprouts. (**G)** *Rictor*^iΔec^ embryos display a delay in ossification. Representative pictures of the upper limbs of embryos stained with alizarin red (bone) and alcian blue (cartilage). Arrows: ossification centers. Below, quantification with number of ossification centers in fingers upon knockout of *Rictor* at indicated starting time points of Tx injections. *P < 0.05, **P < 0.01, compared to E14.5; n_embryo_ = 4 (number of centers was measured in both extremities and averaged for each embryo), Mann-Whitney Rank Sum Test.

Whole-body mTORC2 knockout mice are embryonically lethal. Guertin and colleagues suggested vascular defects as a potential reason for early embryonic death^14^,^15^. We further investigated the loss of *Rictor* in endothelial cells during embryogenesis by using a constitutive VE-Cadherin promoter-driven Cre and LacZ reporter containing^19^ *Rictor* knockout. The analysis of 101 pups revealed two homozygous *Rictor* knockout mice, indicating predominant embryonic lethality. Heterozygous *Rictor* knockout and wildtype mice were born at expected Mendelian ratios (Fig. 1A). Interestingly, the two surviving *Rictor*^Δec^ mice were females, fertile, and phenotypically normal. When further used for breeding, these two *Rictor*^Δec^ mice gave birth only to heterozygous and wildtype *Rictor* pups. We then analyzed 43 embryos received after terminated pregnancy on day E10.5. 11 out of these embryos were genotyped as homozygous knockouts. LacZ reporter-positive *Rictor*^Δec^ and wildtype embryos displayed endothelial cell-specific Cre recombination, as visualized by β-galactosidase staining in intersegmental vessels, intracranial arteries, and the dorsal aorta. *Rictor*^Δec^ homozygous embryos generally appeared normal and were viable at this time point. LacZ activity in the dorsal longitudinal anastomotic vessel was clearly visible in wildtype embryos but was less prominent in 7 out of 11 *Rictor*^Δec^ embryos, indicating reduced or delayed angiogenesis into the periphery from intersegmental vessels (Fig. 1B, for additional pictures see supplementary Fig. S1). We found only one noticeable distinct vascular feature in 2 out of 11 *Rictor*^Δec^ embryos compared to controls: Vascular remodeling around the vitelline artery that usually occurs at this or earlier timepoints^20^ was characterized by the presence of numerous, thin parallel anastomosing vessels which are not observed in wild type mouse embryos (arrows in supplementary Fig. S1, lower panels). Thus, erythropoiesis and vasculogenesis of the primitive vascular plexus with further remodeling of arteries and veins, which is completed on E10.5^21^, was not modulated by endothelial mTORC2. To precisely determine the time point of lethality, overlapping tamoxifen-injection schemes in pregnant mice with homozygous loxed *Rictor* gene and inducible VE-Cadherin CreER^T2^ recombinase were used (Fig. 2A). Injecting tamoxifen three times in pregnant mice, starting at E7.5, produced marked and significant reductions in litter size (Fig. 2B). However, injecting tamoxifen twice at E7.5 did not result in any differences in litter size (Fig. 2B). *Rictor* knockout by 60% is achieved with two injections of tamoxifen, whereas nearly homozygous (92%) knockout is achieved with three injections of tamoxifen every second day^22^. Thus, with three injections starting on E7.5, knockout of *Rictor* was likely to be maximal starting from E11.5–E12.5. On E17.5, one third of the embryos were growth retarded, and the remaining embryos were absorbed (Fig. 2C). In addition, more than 90% of analyzed embryos were growth retarded after tamoxifen injections began on E6.5 and E8.5 (Fig. 2C). Interestingly, tamoxifen injections that began on E12.5 and E14.5 had no influence on viability and growth (Fig. 2C).

Embryos that were injected with tamoxifen on E8.5 had a body length of approximately 14 mm, whereas embryos that were injected on E14.5 had a body length of 19.5 mm. Wildtype embryos at embryonic day 17.5 displayed a body length of 18–22 mm (Fig. 2D). Furthermore, growth-retarded embryos did not display wrinkled skin; instead, the skin was rather thin, and subcutaneous veins were visible (Fig. 2E). To investigate whether endothelial-specific *Rictor* knockout causes a delay in vascularization, embryos received three injections of tamoxifen that started on E7.5, after which they were sacrificed at E12.5. The majority of endomucin-stained vascular plexi were present in both *Rictor*^iΔec^ and control embryos. Due to mortality at this time point, we detected only 5 knockout embryos out of three terminated pregnancies with induced knockout. We detected reduced sprouting angiogenesis in subdermal vessels in the ventral region and of *Rictor*^iΔec^ embryos (2 out of 5 *Rictor*^iΔec^ embryos) (Fig. 2F). Also, the temporary hyaloid vessels in the eye had formed incompletely (3 out of 5 *Rictor*^iΔec^ embryos) (Fig. 2F). In growth-retarded *Rictor*^*i*Δec^ mice that were that were first injected with tamoxifen on E.6.5 and E8.5, ossification centers in the fingers were strongly reduced or missing when analyzed on E17.5 as compared to embryos that were first injected on E14.5 (Fig. 2G, alizarin red (bone) and alcian blue (cartilage) stainings and quantification of ossification centers below). Embryos that were first injected on E14.5 displayed an ossification progress comparable to wildtype mice as demonstrated earlier by Gollner *et al.*^23^ Similarly, the progress of ossification in the vertebrae was clearly delayed in embryos that were first injected with tamoxifen on E8.5, and the long bones of the upper and lower limbs were significantly shorter in embryos that received their first tamoxifen injections on E6.5 and E8.5 compared to embryos that received their first tamoxifen injections on E14.5 (supplementary Fig. S2). CD31 staining from skin, brain, skeletal muscle, lung, and colon sections of growth-retarded *Rictor*^iΔec^ mice on the other hand were morphologically indistinguishable from sections of wildtype control mice (supplementary Fig. S3).

In summary, endothelial *Rictor* knockout resulted in lethality which peaked around E12 and growth retardation close to this time point in midgestation. Growth retardation was accompanied by delayed bone ossification in fingers, toes and vertebrae. *Rictor* knockout delayed peripheral angiogenesis, but did not generally affect vascular plexus formation.

### Endothelial-specific *Rictor* knockout has no obvious effect on viability and weight gain during adolescence into adulthood

**Figure 3:**
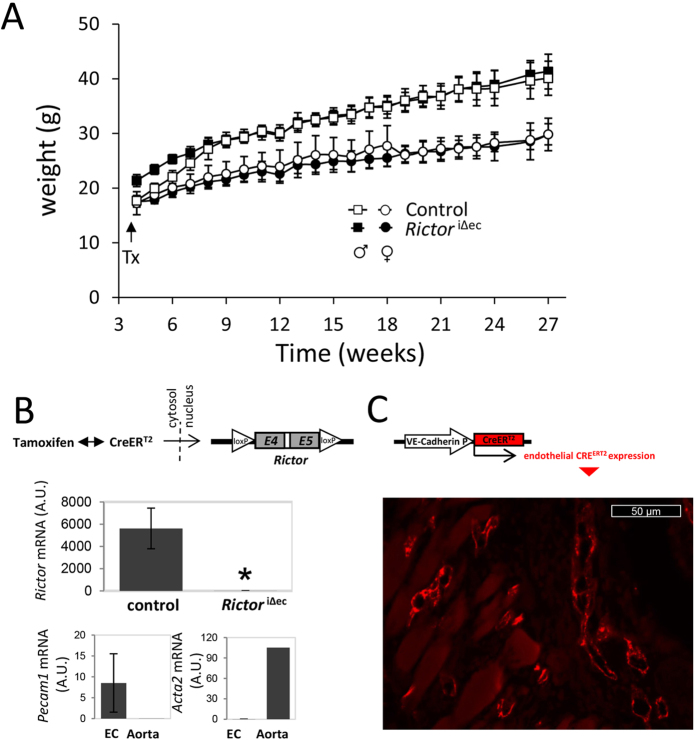
*Rictor* knockout does not affect weight gain and viability in adolescent mice. **(A)** Body weights were followed in male and female mice over a period of 27 weeks after induction of knockout or control on week 4. n = 10 per genotype and gender, n.s., 2-way ANOVA with group-wise comparison. All mice displayed normal health, behavior, and viability. (**B)** At the end of the experiment, RNA was extracted from endothelial layer for quantitative polymerase chain reaction (PCR) analysis to test for efficient excision of *Rictor* (n = 3/2, *P < 0.05. 2-tailed T-test), and qualitatively for purity of endothelial tissue (endothelial marker *Pecam1*, smooth muscle marker αSMA). Total aorta mRNA was used as comparative control. Representative immunostainings for estrogen receptor 2 (red fluorescence) in histological sections of the skinfold from 10-week-old *Rictor*^iΔec^ mice demonstrates specific expression of CreER^T2^ recombinase associated with capillaries.

We proceeded to analyze the general requirement of endothelial mTORC2/RICTOR using the inducible VE-Cadherin-driven CreER^T2^ variant (*Rictor*^iΔec^) during adolescence from the age of 4 weeks (at the time of tamoxifen-induced *Rictor* knockout) to adulthood, up to an age of 28 weeks. No statistically significant differences in weight gain were observed between genotypes and genders (Fig. 3A). Furthermore, all groups displayed normal health and viability. At the end of the weight study, the aorta from male control and *Rictor*^*i*Δec^ mice were removed, the endothelium was scraped, and *Rictor* mRNA levels were determined by quantitative polymerase chain reaction (qPCR) to show stable, efficient, and significant knockdown of *Rictor* (Fig. 3B). Adolescent double transgenic mice (Cre^+/+^; *Rictor*^lox/lox^) also displayed specific CRE expression in capillaries from the mouse dermal skin muscle, as shown by estrogen receptor-specific staining in tissues that were used for intravital experiments (Fig. 3C). Although VE-Cadherin promoter-driven Cre recombination may also affect hematopoietic development^22^, we found similar hematological profiles in 8-week-old control and *Rictor*^iΔec^ mice. These profiles were comparable to those of healthy C57/Bl6 mice (supplemental Fig. S4).

These experiments demonstrate that the stable ablation of endothelial mTORC2 in adolescent mice, which persisted until late adulthood, did not cause obvious general health problems, as supported by normal hematological profiles, weight gain, and viability.

### *Rictor* knockout in mouse aortic endothelial cells (MAEC) differentially disables the formation of capillary-like endothelial networks

**Figure 4:**
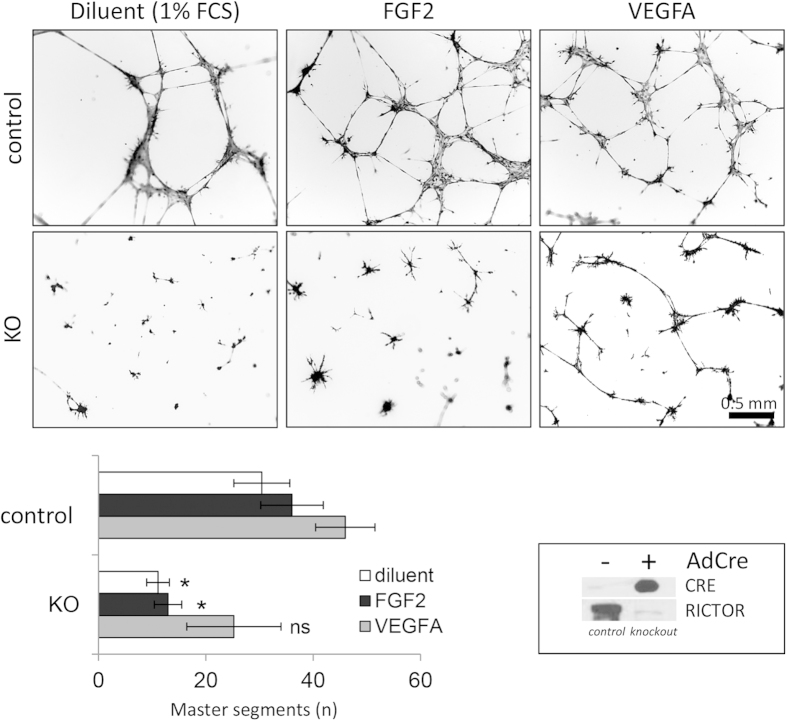
*Rictor* knockout in mouse aortic endothelial cells (MAEC) decreases endothelial network formation. **(A)** Representative micrographs show endothelial network formation after 18 hours of seeding. Quantification (total number of master segments that connect to at least two other segments) of endothelial tube formation from three experiments is shown below. (Bars; mean and SEM, n_exp_ = 3, *P < 0.05 versus control. Paired T-Test). FGF-treated *Rictor* ko MAEC typically formed star-shaped centers with omni-directional sprouting and no connection to neighboring centers. Efficient *Rictor* knockout displayed by RICTOR and CRE protein expression from control (AdCre–) and *Rictor* ko (AdCre+) MAEC (n = 3) is displayed on the lower right.

Our results so far suggested, that knockout of *Rictor* in endothelial cells has no obvious effects on the development during adolescence in mice and therefore may not affect basic physiologic parameters of the endothelial cell such as survival and homeostatic functions in the existing and developing vasculature. Before continuing investigations about the role of mTORC2 in activated endothelium *in vivo*, we used an *in vitro* assay to determine the angiogenic response of control and *Rictor* knockout mouse aortic endothelial cells to the two major angiogenic molecules FGF and VEGF (Characteristic endothelial markers of these cells are shown in supplementary Fig. S5). After plating on a basement membrane matrix gel, endothelial cells build capillary-like tubes with a lumen within a short time. Cells initially attach to the matrix and then migrate towards each other, after which they align and form tubes^24^. We found that control MAEC formed connected master segments in the presence of the diluent (1% fetal calf serum [FCS]), FGF2, and VEGFA (Fig. 4A). Rictor knockout was induced by CRE recombinase expression after adenoviral transfection (Fig. 4, lower left panel). We found, that the ability of MAEC with *Rictor* knockout (*Rictor* ko MAEC) to build stable contacts and connecting tubes was substantially disabled: Knockout of *Rictor* prevented MAEC from forming capillary-like tubes in unstimulated conditions. Upon FGF2 stimulation, *Rictor* ko MAEC arranged into star-like shapes, with sprouts extending from cell clusters, but did not establish contacts to other cell clusters and formed significantly less master segments compared to control (Fig. 4). In contrast to the study by Wang *et al.*^17^, VEGFA stimulation partially rescued endothelial network formation in *Rictor* ko MAEC. *Rictor* ko MAEC were able to form some substantial networks and organized tubes, albeit in in numbers that were small and similar to those of control MAEC (Fig. 4).

As we found that the angiogenic response to FGF2 was significantly more affected by *Rictor* knockout than that to VEGF, we further focused on FGF2-mediated responses in endothelial cells and angiogenesis assays *in vivo* in this study.

### FGF2 amplifies RICTOR protein and RICTOR-dependent phosphorylation of AKT on serine 473 and PKCa on serine 657

**Figure 5:**
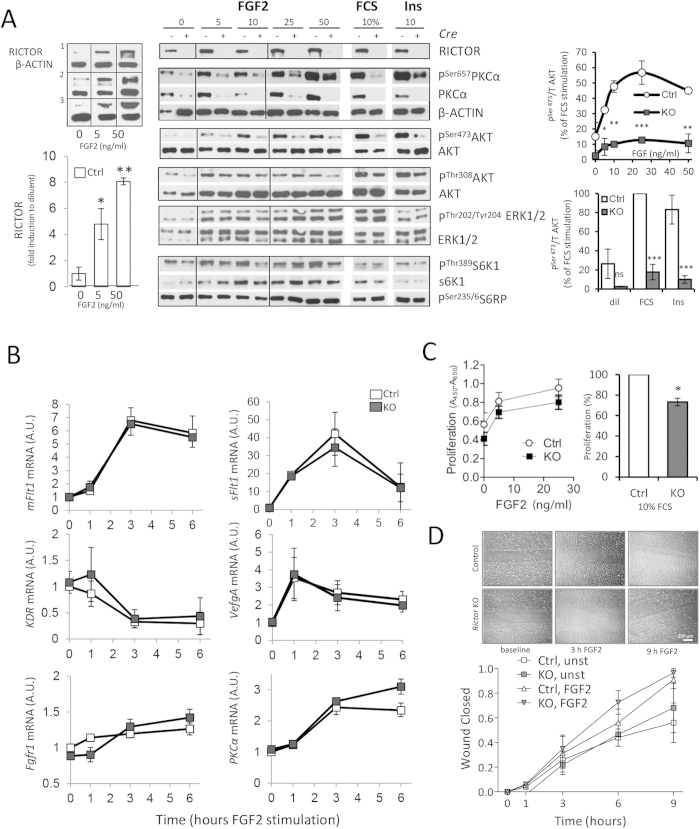
FGF2 amplifies RICTOR protein and *Rictor*-dependent phosphorylation of AKT on serine 473 and PKCa on serine 657. (**A**) Western blots show RICTOR and downstream targets of mTORC2 after 15 min stimulation of control and *Rictor* ko MAEC with 5–50 ng/ml of FGF2, 10% FCS or 1 μg/ml insulin (Ins). Significant increase in RICTOR protein at 5 ng/ml of FGF2 stimultion peaking at an 8-fold expression at 50 ng/ml of FGF2 compared to diluent in 3 repeated experiments in a MAEC isolate (control = white bars, n_exp_ = 3, *P < 0.05, **P < 0.001, 1-way ANOVA with Bonferroni multiple comparison, upper left panels). PKCα protein was nearly absent in *Rictor* ko MAEC compared to control (middle panels). Densitometric quantification shows dose-dependent AKT ^Ser473^ phosphorylation in response to FGF2, FCS and Ins (right panels, n_exp_ = 3, *P < 0.05, **P < 0.01, ***P < 0.001, repeated measures ANOVA). Lower middle blots show S6K1 phosphorylation on P^Thr389^ compared to total S6K1 and phosphorylation of ERK1/2 on P^Thr202/Tyr204^ compared to total ERK1/2 after *Rictor* knockout. (**B)** mRNA expression after 1–6 hours stimulation (25 ng/ml FGF2) of control and *Rictor* ko MAEC of VEGF receptor 1 (*mFLT1, sFlt1*), VEGF receptor 2 (*Kdr*), VEGFA (*Vegfa*), FGF receptor 1 (*Fgfr1*) *and* protein kinase Cα (*PKCα*) detected by quantitative real-time PCR (n = 3, ns knockout versus control, repeated measures ANOVA). (**C)** Absolute proliferation values (Absorption = A4_50 nm_-A_650 nm_) in FGF2-stimulated control (open circles) or *Rictor* ko (filled squares) MAEC are presented. (n_exp_ = 3, n.s., repeated measures ANOVA). In response to 10% FCS (right), proliferation of *Rictor* ko MAEC was significantly lower compared to that of control MAEC (n = 3, *P < 0.05, two-tailed T-test). (**D)** Migration of MAEC was measured after FGF2 (25 ng/ml) or diluent administration for 1, 3, 6 and 9 hours (wound completely closed = 1, n = 3, n.s., repeated measures ANOVA).

Interestingly, we found a consistently low expression of RICTOR in starved, unstimulated and confluent MAEC isolates, whereas FGF2 amplified RICTOR protein levels. Quantification demonstrated a significant increase in RICTOR protein at 5 ng/ml of FGF2 peaking at an 8-fold expression compared to diluent at 50 ng/ml of FGF2. (Fig. 5A, upper left panels). We assessed how FGF2-induced signaling is altered in mTORC2-deficient endothelial cells and focused on one of the main mTORC2 downstream targets, PKCα and AKT^25^. In control MAEC, FGF2 induced the dose-dependent phosphorylation of the hydrophobic motif (HM) of PKCα on serine 657 (P^Ser657^PKCα). Interestingly, deletion of *Rictor* strongly decreased PKCα protein levels and accordingly blunted the phosphorylation of the HM of PKCα in response to FGF2, FCS, and insulin (Fig. 5A). PKCα signaling was strongly disabled in *Rictor* ko MAEC, and this was most likely a result of total PKCα protein destabilization and degradation due to absent phosphorylation^25^. We then assessed the impact of *Rictor* deletion in MAEC on mTORC downstream target protein kinase AKT (also known as PKB). The phosphorylation of the activation loop (A-loop) on threonine 308 (P^Thr308^AKT) by phosphoinositide-dependent kinase 1 (PDK1) and of the HM at serine 473 of AKT (P^Ser473^AKT) by mTORC2 results in AKT activation^11^,^26^. In control MAEC, FGF2 induced a marked and dose-dependent P^Ser473^AKT that peaked at 25 ng/ml of FGF2. *Rictor* knockout efficiently and significantly blunted FGF2-, FCS-, and insulin-induced AKT phosphorylation (Fig. 5A, quantification to the right). The disruption of mTORC2 has been shown to decrease P^Thr308^AKT in some cancer cell lines^11^. In control MAEC, P^Thr308^AKT was robustly induced by FGF2, FCS, and insulin. After *Rictor* knockout, we did not detect a significant reduction in P^Thr308^AKT (Fig. 5A). These results are consistent with reports in cells or tissues that were derived from mice with genetic deletions of mTORC2 components. In these mice, A-loop phosphorylation was not disrupted in the absence of P^Ser473^AKT^14^,^25^,^27^.

The depletion of AKT phosphorylation by *Rictor* knockout may influence mTORC1 signaling. AKT phosphorylates and inhibits tuberous sclerosis 2 (also known as tuberin), thus resulting in the activation of mTORC1- p70S6 kinase1 (S6K1)^28^. We found that FGF2 dose-dependently promoted S6K1 phosphorylation in control MAEC. After *Rictor* knockout, a minor but insignificant reduction in FGF2-induced S6K1 phosphorylation was detected (Fig. 5A). No differences in the phosphorylation of extracellular signal-regulated kinase 1 and 2 (ERK1/2) by the FGF2 gradient were observed in control MAEC and *Rictor* ko MAEC (Fig. 5A)^29^.

A close cross-talk exists among FGF2 and the different members of the VEGF family during angiogenesis and several studies have suggested that FGF2 induces neovascularization indirectly by activation of the VEGF/VEGFR system^30^. We confirmed that *Vegfa* mRNA substantially increased after FGF2 stimulation, yet *Rictor* knockout did not affect this induction. To investigate whether endothelial mTORC2 might modulate *in vitro* angiogenesis in response to FGF2 by regulation of VEGF receptors, we measured the mRNA-levels of the two main VEGF-receptors *Flt1* (VEGFR1), which is characteristic for stalk cells and quiescent endothelium, and *Kdr* (VEGFR2), upregulated in tip cells^31^. Interestingly we found a strong FGF2-induced upregulation of the membrane form of *Flt1* (*mFlt1*) and an even stronger induction of soluble Flt1 receptor (sFlt1) mRNA (Fig. 5B). *Kdr* message was present and decreased after FGF-stimulation (Fig. 5B). However, *Rictor* knockout cells induced *mFlt1* and *sFlt1* and reduced *Kdr* message congruent to control cells after FGF2 stimulation, which argues against endothelial tip-/stalk-cell modulation by mTORC2. FGF2 also stimulates VEGFA in endothelial cells of forming capillaries and cultured aortic endothelial cells^32^. Similarly, FGF receptor 1 (*Fgfr1*) message equally increased in control and *Rictor* ko MAEC. The decrease in PKCα protein after *Rictor* knockout was due to posttranscriptional mechanisms as PKCα gene expression was equal in control and *Rictor* ko MAEC (Fig. 5B).

Furthermore, we did not observe any significant differences in endothelial proliferation over a period of 72 hours in the dose response to FGF2 (Fig. 5C) or FGF2-induced-migration in a wound assay (Fig. 5D). However, a weak but significant (P < 0.05, N_exp_ = 4) reduction in proliferation was detected in the presence of FCS (Fig. 5C). Similarly, we found a significant reduction in VEGFA-induced proliferation by *Rictor* knockout (Supplemental Fig. S6).

In conclusion, FGF2 amplified RICTOR protein in control MAEC and *Rictor* deletion in FGF2-stimulated MAEC strongly blunted PKCα signaling and reduced AKT activity by depleting Ser473 phosphorylation. *Rictor* deletion had no effects on mTORC1 signaling and FGF-induced proliferation, migration nor did it interfere with FGF2-induced modulation of *Flt1*, *Kdr* and *Vegfa* expression levels.

### The structure of the capillary bed of the striated skin muscle is not altered by endothelial-specific *Rictor* knockout

**Figure 6:**
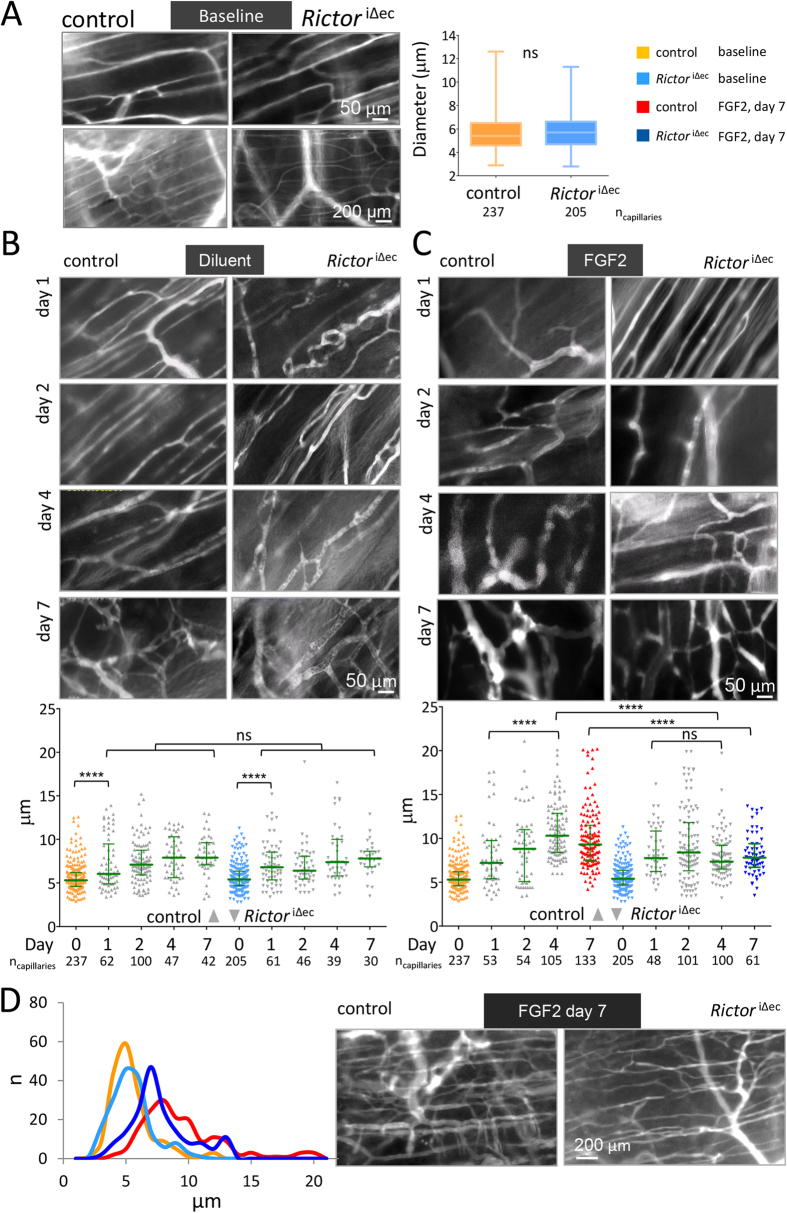
*Rictor* knockout disables a sustained increase in skin capillary diameters and restricts extensive capillary remodeling in response to FGF2. (**A)** Stacked frames of representative videos of the capillary vasculature were recorded through the dorsal skinfold chamber by intravital fluorescence microscopy. Baseline capillary bed of the skin muscle was assessed in 10-week-old mice (6 weeks after knockout induction) by intravital microscopy through the unmodified dorsal skinfold chamber. Upper (20 × magnification) and lower micrographs (10×) display representative skin capillary beds of control (left) and *Rictor*^iΔec^ (right) mice. Capillary diameters from 10 control and *Rictor*^iΔec^ mice were quantified and pooled for statistical analysis (n_capillaries_ = 253/206, no differences between groups, 2-tailed T-test and Whisker plot, indicating median, 25% percentile and total range to the right). Color scheme applies for whole Fig. 6 and is displayed in upper right corner. **B.** Wounding response of the capillary bed. Skin muscle capillary structure from day 1, 2, 4 and day 7 in control (left) and *Rictor*^iΔec^ mice (right) after wound sealing with heparin-containing matrigel (20 × magnification). Capillary diameters from 4 control and *Rictor*^iΔec^ mice were quantified and pooled for statistical analysis by 1-way ANOVA followed by Bonferroni multiple comparison (Scatter plot with medians on the below; number of capillaries (n_capillaries_) are indicated below the X-axis, ****P < 0.0001). **C.** Heterogeneous capillary diameter increase in the FGF2-stimulated capillary bed. Skin muscle capillary structure from day 1, 2, 4 and day 7in control (left) and *Rictor*^iΔec^ mice (right) after wound sealing with FGF2 (1.5 μg/ml; heparin-containing matrigel, 20 × magnification). Capillary diameters from 7 control and *Rictor*^iΔec^ mice were quantified and pooled for statistical analysis by 1-way ANOVA followed by Bonferroni multiple comparison (Scatter plot and medians below; number of capillaries are indicated below the X-axis, ****P < 0.0001). **D.** Capillary remodeling in the FGF2-stimulated capillary bed. The line graph displays the normalized distribution of capillaries (n_max_ = 100) resolved in a 1-μm range after FGF2 stimulation for 7 days and illustrates differences in remodeling between groups. Micrograph to the right shows 10x magnification of the vascular bed of control and *Rictor*^iΔec^ mice after 7 days of FGF2 exposure.

In preparation to study mTORC2-dependent vascular changes in response to wounding and FGF2 *in vivo*, we first recorded the capillary morphology in the existing striated skin muscle (*Panniculus carnosus*) vascular bed as baseline. To do so we surgically mounted a dorsal skinfold chamber^33^,^34^ to 8-week-old control and *Rictor*^iΔec^ mice. We observed that the baseline capillary bed, which was recorded 3 days post-chamber surgery, appeared very similar in 8-week-old control and *Rictor*^iΔec^ mice (Fig. 6A; supplementary video 1). Analysis of all capillary diameters measured at baseline did not reveal significant differences (Fig. 6A)). In both experimental groups, the vascular bed consisting of the *panniculus carnosus* with its capillary structures was organized in the typical parallel orientation, and the subcutaneous layer with draining arterioles and venules exhibited the typical perfusion pattern of the dorsal skinfold chamber capillaries, as observed in previous studies^33^. Thus, ablation of endothelial *Rictor* did not interfere with the development or directly observable functionality of the intact, unstimulated dermal microvasculature in adolescent mice, which is in line our observation of normal growth, viability and weight gain of adolescent mice lacking endothelial *Rictor*.

### Wounding-induced capillary diameter remodeling is not impaired by endothelial-specific *Rictor* knockout

We then modified this chamber by additionally removing cutis and subcutis on the opposite side of the observation window. This defect was sealed by growth factor-reduced matrigel (suppl. Fig. S7). In the first set of experiments, we included diluent (heparin) in matrigel to assess baseline alterations as a response to wound healing processes without exogenous growth factor stimulation. Capillaries were observed and recorded on a daily basis by intravital microscopy up to 7 days after matrigel sealing (Fig. 6B, and supplementary video 2). Vascular morphological parameters were quantified in vessels where perfusion was observable. The vessels encompassed diameters ranging from 4 to 20 μm, thus capillaries and some small arterioles or venules. Capillary diameters significantly increased from day 1, 2, 4 and 7 compared to the baseline by about 2 μm in average for both control and *Rictor*^iΔec^ capillary diameters (Fig. 6B). Importantly, the microvasculature of the *panniculus carnosus* responded similarly to wounding and matrigel sealing in control and *Rictor*^iΔec^ mice as we did not detect significant differences compared to controls at days 1, 2, 3, and 7 (Fig. 6B).

### Endothelial-specific *Rictor* knockout limits increases in the capillary diameter of existing skin vasculature in response to high doses of FGF2

To assess remodeling in terms of individual increases in capillary diameters in response to strong angiogenic stimulation, we exposed the existing capillary bed to a high dose of FGF2 (1.5 μg/ml). We focused on FGF2, since we found that *Rictor* deletion specifically disabled FGF2- but not VEGFA-dependent angiogenesis *in vitro* as shown earlier in this manuscript. Furthermore, FGF2 also promotes maturation of larger vessels such as arterioles^35^. Intravital recordings through the dorsal skinfold chamber in control and *Rictor*^iΔec^ revealed that diameters increased at day 1 and day 2 after FGF2 exposure (Fig. 6C, day 1 and 2). In both groups, diameters had increased for about 3.5 μm and we found no statistical difference between groups after testing by Bonferroni multiple comparisons (Fig. 6C). At days 4 and 7, capillary diameters further significantly increased in the control group when comparing to diameters at day 1. In addition, tortuous and bulbous vascular structures developed, which were observed in capillaries and small draining arterioles and venules (Fig. 6C, day 4 and 7). In *Rictor*^iΔec^ mice, however, FGF2-induced capillary enlargement stopped and rather regressed after day 2. Statistically, capillary diameters at days 4 were not different from those measured at day 1. Diameters measured on day 4 and 7 in *Rictor*^iΔec^ were significantly smaller compared to the control group on those days (Fig. 6C). We observed, that at the end of the recording the vascular bed in *Rictor*^iΔec^ mice underwent a far more restrained and different mode of remodeling. Thin-connecting anastomoses emerged between capillaries and draining arterioles in *Rictor*^iΔec^ mice, and the orientation of capillaries remained largely parallel (Fig. 6C and D, day 7 and supplementary video 3 and 4). The dilated and tortuous capillary structures that developed in control mice after longer (4 and 7 days) exposure to FGF2 are also found in vascularized tumors, which in addition exhibit hyperpermeability^36^. We assessed vessel leakage of the FGF2-stimulated skin vascular bed on day 7 by intravenously injecting fluorescently labeled ricinus communis agglutinin I (RCA I)^37^. Preliminary data could however not support an obvious decrease in permeability by endothelial *Rictor* deletion as plasma leakage points were present in capillary structures from both control and *Rictor*^iΔec^ mice to a similar extent (suppl. Fig. S8).

Taken together, loss of endothelial *Rictor* normalized vascular structure after 4 days of exposure to FGF2 and prevented the formation of a heterogeneous, irregular microvascular bed as seen in controls (Fig. 6D). This suggests that endothelial mTORC2 is central to the FGF2-mediated remodeling response that creates larger and heterogeneously sized capillaries and small arterioles in the existing skin capillary bed beyond day 4.

### Endothelial-specific *Rictor* knockout strongly reduces FGF2-induced neovascularization in matrigel plugs and prevents local hemorrhage

**Figure 7:**
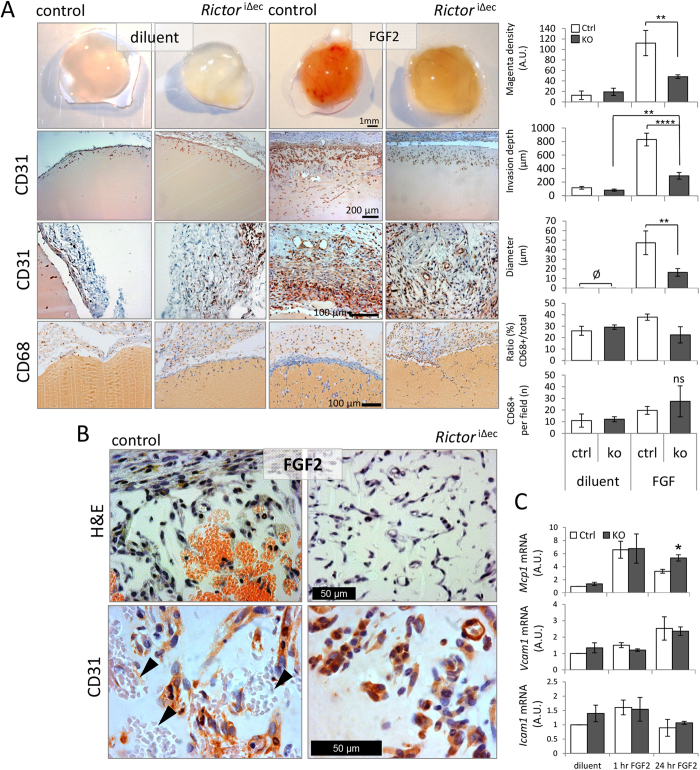
Fibroblast growth factor 2(FGF2)-induced angiogenesis in matrigel plugs is reduced in *Rictor*^iΔec^ mice. (**A**) 1^st^ row: Representative matrigel plugs containing heparin (diluent) or 1.5 μg/ml FGF2 with heparin (FGF2) from 8-week-old male control (left) and *Rictor*^iΔec^ (right) mice removed 7 days after injection (scale bar = 1mm). Estimation of blood content by optical densitometry is shown on the right (n_plugs_ = 4; **P < 0.01; 1-way ANOVA with Bonferroni multiple comparison). (**A**) 2nd row: Paraffin sections from corresponding plugs immunostained for CD31 (brown) and hematoxylin (blue/nuclei). Representative micrographs show 10 × magnification and display the depth of newly in grown microvessels from the surface towards the center of the matrigel plugs. Quantification to the right displays the significant reduction in the ingrowth (μm) of neovessels into plugs from *Rictor*^iΔec^ mice compared to control mice (n_plugs_ = 4/7; **P < 0.01, ****P < 0.0001; 1-way ANOVA with Bonferroni multiple comparison). (A) 3^rd^ row: Representative micrographs of peripheral stroma covering matrigel plugs. Identifiable inner microvessel diameters were measured (graph to the right; n_plugs_ = 3; *P < 0.05;2-tailed T-test ). No vessels were found in peripheral stroma covering diluent-containing plugs. (**A**) 4^th^ row: Representative micrographs of macrophage marker CD68-immunestainings of peripheral stroma and matrigel. Graphs to the right show ratio of CD68^+^/total cell nuclei in the stroma, and average of CD68^ + ^cells per field counted in the stroma (n_plugs_ = 4, no significant differences found after 1-way ANOVA/Bonferroni multiple comparison). (**B)** Representative micrographs of one set of experiments displaying hematoxylin and eosin stained (H&E) matrigel areas showing local leakage and hemorrhagic areas in FGF2 containing plugs from control mice compared to plugs from *Rictor*^iΔec^ mice (upper micrographs). Lower micrographs show higher magnification of CD31-stained matrigel areas. Arrowheads point to local spots of leaked erythrocytes in FGF2-containg control plugs. (**C)** Confluent and starved control and Rictor ko MAEC were stimulated for 1 and 24 hours with 25 ng/ml FGF2 or diluent. mRNA expression of monocyte attracting protein 1 (*Mcp1*), vascular and inducible cell adhesion molecules 1 (*Vcam1, Icam1*) was detected by quantitative real-time PCR (n = 3; *P < 0.05; 1-way ANOVA with Bonferroni multiple comparison test).

As technical limitations disabled us to monitor sprouting angiogenesis in the dorsal skinfold chamber, we used the matrigel plug angiogenesis assay to assess whether endothelial mTORC2 deficiency may alter *de novo* vascularization in adult mice *in vivo* in response to strong stimulation by FGF2 (1.5 μg/ml). Concentrations of FGF2 in this range have been used previously to achieve maximal neovascularization after 7 days^38^ and to achieve macroscopically a hemorrhagic appearance (see also supplementary Fig. S9). Knockout of *Rictor* in the endothelium was induced during adolescence in *Rictor*^iΔec^ mice. At 7 days after implantation, blood-containing microvessels and signs of hemorrhage were macroscopically evident in FGF2-containing plugs from control mice. In contrast, FGF2-containing plugs from *Rictor*^iΔec^ mice displayed homogeneous, unobtrusive vascularization. An estimation of blood content by optical densitometry demonstrated a significant decrease after *Rictor* knockout in FGF2 containing plugs compared to control plugs (Fig. 7A, 1^st^ row). FGF2-induced vessels protruded to about 800 μm from the surface of the plug into the center in plugs from control mice, whereas microvessels protruded significantly less to only about 300 μm in plugs from *Rictor*^iΔec^ mice. FGF2-induced microvessel protrusion into the plugs from *Rictor*^iΔec^ mice was not completely abolished as we calculated a significant difference to diluent containing plugs (Fig. 7A, 2^nd^ row). The presence of leaked erythrocytes in the FGF2-containing plugs from control mice from one set of experiments demonstrated local hemorrhage (Fig. 7B, left micrographs). In contrast, the microvasculature in FGF2-containing plugs from *Rictor*^iΔec^ mice was composed of homogenously small capillaries with no evidence of hemorrhage (Fig. 7B, right micrographs). Further micrographs showing hemorrhage in FGF2-containing plugs from 2 other sets of experiments are displayed in supplementary Fig. S10. During the 7 days of FGF2 administration, the matrigel plug was encapsulated by a thin layer of stromal tissue that also contained arterioles and venules. We found similar vessel densities in plugs from both experimental groups (data not shown), whereas the luminal diameter was significantly smaller in vessels of the stromal capsule from *Rictor*^iΔec^ mice compared with control mice. No stromal vessels were found in diluent containing plugs from both groups (Fig. 7A, 3^rd^ row). Angiogenesis occurring during postnatal development is usually connected with inflammation^39^. Macrophages were demonstrated to promote angiogenesis via FGFs and placental growth factor signaling^39^ or by the release of pro-angiogenic molecules^39^,^40^. We therefore investigated by CD68 immunestaining whether the stromal halo around the plugs may contain varying amounts of macrophages that could influence *de novo* angiogenesis in our experimental setting. However, we found no significant differences in the ratio between CD68^+^ cells to total cell nuclei in the peripheral stroma when comparing diluent and FGF2-containing plugs from both groups (Fig. 7A, last row). The total amount of CD68^+^ cells per field counted was also not significantly modulated, however, we noticed a trend towards higher macrophage count in stromal halos around FGF2-containing matrigel plugs from *Rictor*^iΔec^ mice (Fig. 7A, last row). To further investigate the possibility of a mild FGF2-dependent proinflammatory state after endothelial *Rictor* knockout we measured the mRNA levels of monocyte-attracting protein 1 (*Mcp1*) and vascular- and inducible cell adhesion molecules (*Vcam1, Icam1*) in MAEC. FGF2 increased *Vcam1* mRNA after 24 hours similarly in both control and *Rictor* knockout MAEC. A slight and insignificant increase for *Icam1* mRNA was observed. FGF2 robustly (ca. 6.6 fold) induced *Mcp1* mRNA in control and *Rictor* knockout MAEC. Interestingly, *Mcp1* mRNA remained at high levels (ca. 5.3 fold induction) after 24 h of FGF2 stimulation and was significantly higher compared to controls (3.3 fold) at this timepoint (Fig. 7C). Thus, it is unlikely, that the strong reduction of FGF2-induced angiogenesis into matrigel plugs along with ‘normalized’ microvessel features by deletion of endothelial *Rictor in vivo* is caused by an altered inflammatory response.

Taken together, we observe a dense, heterogeneous neovasculature with several patches of hemorrhage indicating disrupted or leaky capillaries that formed after 7 days of FGF2-exposure in control mice. In contrast endothelial *Rictor* knockout strongly and significantly reduced neovessel ingrowth in response to FGF2 with capillaries remaining homogenously small, with no evidence of hemorrhagic spots.

## Discussion

In this study we deleted *Rictor* in the endothelium to study the general requirement of endothelial mTORC2 during embryonic and adolescent development. Our analysis of embryonic development using constitutive and inducible VE-Cadherin-Cre-specific *Rictor* knockout confirmed that homozygous *Rictor* deletion in endothelial cells is almost completely lethal and results in embryonic death around E12. Guertin *et al.* proposed that vascular defects may contribute to the lethality of whole-body *Rictor* knockout embryos on E10.5^14^. Furthermore, Wang *et al.* demonstrated that the homozygous and Tie2-Cre-driven *Rictor* knockout is embryonically lethal^17^. Still, other than reductions in peripheral vascularization, we did not detect gross abnormalities of the normal vascular plexi on E10.5. Surviving embryos around this time frame showed rather distinct and rare deficits in vascularization at E10.5. Surviving Rictor knockout embryos, however, consistently displayed growth retardation and a delayed ossification of the vertebrae, toes, and fingers. Delayed ossification may explain growth retardation but may not categorically explain embryonic lethality of *Rictor*^iΔec^ in the confined midgestational timeframe. Thus, other essential functions that are regulated by mTORC2 during this short period in midgestation are probable and may also account for lethality.

Many signaling pathways that are involved in early embryonic development are also recapitulated during tumorigenesis^41^. As we found that endothelial mTORC2 was required during a confined, midgestational timeframe but had no apparent influence on viability before midgestation (E8.5), beyond midgestation (E14.5), physiological vascular development, vascular maintenance and growth from adolescence into adulthood, we hypothesized that endothelial *Rictor* might promote only ‘aberrant’ endothelial phenotype modulation such as in tumor angiogenesis or during a transition to an invasive mesenchymal phenotype. Guertin *et al.* demonstrated that *Rictor* has no significant role by itself in maintaining the integrity of a normal prostate epithelium *in vivo* but requires *Rictor* to be transformed into an invasive, malignant phenotype by *Pten* deletion, which results in strong PI3K activation^42^. Similarly, *Drosophila* embryos lacking mTORC2 activity are viable and display only minor growth defects^42^,^43^. However, *PTEN* loss-induced tissue overgrowth in the *Drosophila* eye requires dTORC2^43^.

We found that particularly FGF2 and not VEGFA depended on the mTORC2 signaling hub to establish a capillary-like endothelial network on matrigel substrate when we tested these PI3K-activating angiogenic molecules *in vitro*. We found that FGF2 elicited a strong and fast increase in the diameter of existing skin capillaries in the dorsal skinfold chamber after two days of stimulation in both control and *Rictor*^iΔec^ mice. Over a period of 7 days, FGF2 induced the progressive and extensive remodeling of vessel structures characterized by heterogeneous and larger diameter sizes that also included larger arterioles and a tortuous vascular bed in control mice. *Rictor*^iΔec^ mice however, could not maintain heterogeneous capillary size remodeling beyond day 2of FGF2 exposure and returned to ‘normalized’ vascular features with homogenously and smaller sized capillary diameters and parallel-oriented capillaries. Tortuous and dilated capillaries are also found in vascularized tumors. Tumor blood vessels furthermore display hyperpermeability, and do not mature into functional vasculature^1^,^2^. However, we could not detect fewer leakage points in FGF2-stimulated skin capillaries *Rictor*^iΔec^ in preliminary investigations.

However, the supply of FGF2 to the skin muscle over a period of seven days was limited by the volume of the matrigel carrier (16 μl) and may not have provided saturated FGF2-stimulation at all times. We therefore continued our investigations using the matrigel plug assay carrying pathologically high doses of FGF2 in a volume of 200 μl to induce aberrant, leaky and tumor-like angiogenesis as reported before^38^. Indeed, we observed a dense, heterogeneous neovasculature with several patches of hemorrhage that formed after 7 days in control mice. Endothelial *Rictor* knockout strongly and significantly reduced FGF2-mediated neovessel ingrowth, and capillaries remained homogenously small with no microscopic signs of leakage. Our study thus demonstrates that endothelial mTORC2 acted as a central signaling hub for FGF2-induced persistent capillary diameter increases and remodeling into heterogeneous capillary structures and formation of a leaky, tumor-like neovasculature in the adult mouse.

FGF2, which is not secreted through vesicular pathways, can be exported from cells with unique extrusion pathways or large amounts can be released upon cell death^44^,^38^. FGF2 has been selectively determined as a crucial tumorigenic cytokine in prostate cancers in which both FGF2 and FGF2 receptor subtypes are co-expressed^45^,^46^. In addition many other tumor types express FGF2/FGFRs at high levels^38^,^47^,^48^,^49^,^50^,^51^. On the other hand, fibroblast growth factors are involved in the formation of skeletal elements within the developing limb^52^,^53^,^54^. Several FGFs are expressed in developing endochondral bone. FGF2 was the first FGF ligand to be isolated from growth plate chondrocytes^52^. FGF2 expression has also been observed in periosteal cells and in osteoblasts^51^,^55^. Targeted deletion of FGF2 causes a relatively subtle defect in osteoblastogenesis, leading to decreased bone growth and bone density^51^,^56^.

Mechanistically, we could not substantiate, that the extensive mTORC2-dependent FGF-induced angiogenic responses were executed by coupling to the VEGFA/VEGFR system, which also determines endothelial tip-/stalk phenotypes^31^. Also, FGF2-induced endothelial proliferation and migration were unaltered after *Rictor* knockout. In contrast, we found VEGFA-induced proliferation to be significantly decreased by *Rictor* knockout as previously reported^17^. Endothelial *Rictor* knockout resulted in dephosphorylation of typical mTORC2 downstream targets and blunted FGF2-induced phosphorylation of P^Ser473^AKT and P^Ser657^PKCα^25^. The results by Wang *et al.* suggest that mTORC2 is a critical signaling node required for VEGF-mediated angiogenesis through the regulation of AKT and PKCα in vascular endothelial cells^17^. AKT is activated by most endothelial growth factors (FGF2, VEGF, and angiopoietin) and orchestrates a number of signaling pathways that are involved in angiogenesis^1^,^57^,^58^. For example, FGF2-induced capillary morphogenesis via FGFR1 is impaired in murine brain capillary endothelial cells expressing an inactive AKT^57^,^59^. FGF2-induced angiogenesis may partially depend on AKT signaling, potentially via transcription factors FoxO1/FoxO3a^27^,^60^.

Interestingly we found that quiescent and starved MAEC expressed low levels of RICTOR in control cells. High doses of FGF2 however induced RICTOR protein in correlation with high P^Ser473^AKT. Comparable findings were reported during the epithelial to mesenchymal transition (EMT). Transforming growth factor (TGF-β), a strong inducer of EMT, increased RICTOR protein and thereby formation of mTORC2 in mouse mammary gland epithelial cells^13^. Without Rictor, the epithelial cells arrested in an intermediate stage between epithelial and mesenchymal differentiation, without the motile and invasive behavior of cells after EMT^13^. Interestingly, TGFβ-1 treatment also induces an interaction between RICTOR and integrin-linked kinase (ILK) and promotes ILK-dependent EMT. This complex was detected in cancer but not in normal cell types^61^ and overlaps with mTORC2 in the function as P^Ser473^AKT kinase^62^. Thus, Rictor could promote EMT by forming different complexes.

In parallel to EMT, endothelial to mesenchymal transition (EndoMT) can be induced by transforming growth factor (TGF-β)^63^. EndoMT is a newly recognized type of cellular transdifferentiation that participates in development, but also in pathological conditions such as cancer and fibrosis^63^,^64^,^65^. New studies have shown that EndoMT represents a dedifferentiation of endothelial cells to a stem cell phenotype, which can redifferentiate into bone or cartilage cells^63^,^64^,^66^. Thus, hypothetically, endothelial Rictor may be required during midgestation to promote the transition of endothelial cells to a mesenchymal, osteogenic phenotyope to promote FGF2-directed ossification of limbs and vertebrae. During further development through normal adolescence where most vasculature is quiescent, with only 0.01% of endothelial cells undergoing division^67^, endothelial *Rictor* knockout may represent the characteristics of an unchallenged quiescent endothelial monolayer with low RICTOR expression and AKT activity. Recent studies suggest, during angiogenic sprouting, endothelial cells express many of EndoMT-driving genes and break down basement membrane. However, they retain intercellular junctions and migrate as a connected train of cells^65^. This process has been termed a partial EndoMT^65^. The permanently activated phenotype of tumor vasculature may well reflect the chronic activation of the EndoMT process, driven by persistent angiogenic cascades, leading to excessive sprouting and a failure to settle back into the mature, stable phenotype^65^. Thus, hypothetically, endothelial mTORC2, assembled through increased expression of FGF2-induced RICTOR may promote neovascularization by a sustained partial EndoMT.

In conclusion, we demonstrated that endothelial *Rictor* is crucial for progression through midgestation and for timely ossification. In adolescence, the FGF2-RICTOR axis promoted sustained, extensive and aberrant neovascularization. During adolescent vascular quiescence or moderate capillary remodeling, however, endothelial mTORC2/RICTOR was not required. Further studies are needed to uncover the exact molecular nature that may enable endothelial mTORC2/RICTOR to promote both embryonic development and extensive and aberrant FGF2-dependent angiogenesis in the adult.

## Materials and Methods

### Animal procedures

Mice with floxed *Rictor* exons^68^,^69^ were crossed with mice that express tamoxifen inducible Cre-ER^T2^ under the control of the endothelium-specific VE-cadherin promoter (VECad-Cre-ER^T2^)^22^ (kind gift of Dr. Iruela-Arispe, Department of Molecular, Cell & Developmental Biology, UCLA, USA) both on a congenic C57Bl/6J background. Offspring were genotyped for *Cre*-recombinase, *Rictor*^floxed^ and *Rictor*^wt^ alleles using qPCR. Briefly, DNA was isolated from ear biopsies and amplified by qPCR with the following primer pairs (5′-3′): Forward: GCG GTC TGG CAG TAA AAA CTA TC; Reverse: GTG AAA CAG CAT TGC TGT CAC TT. The mice were bred, housed and handled according to the local animal ethics committee. All procedures with mice were approved by the Veterinary Office of the Canton of Zürich, Switzerland under licenses 77/2009 and 179/2012. We confirm that all procedures with mice were performed according the legislation of the Swiss Protection of Animals Act for vertebrates within the strict guidelines of the licenses 77/2009 and 179/2012 and additional local directives of the animal housing facilities.

### Whole mount embryo staining

Embryos were stained with an antibody against endomucin according to Vieira *et al.*^70^. In brief, embryos were fixed for two hours on ice with PBS containing 4% PFA. After three washing steps with PBS containing 0.1% Triton X-100 and blocking of unspecific binding sites by incubation with PBS containing 10% FCS and 0.1% Triton X-100 for 30 minutes at room temperature, embryos were incubated with rat monoclonal antibody against endomucin (1:100, Santa Cruz Biotechnology) dissolved in PBS over night at 4 °C followed by five washing steps with PBS at room temperature. After incubation with secondary antibody anti-rat Alexa Fluor-555 (1:200, Molecular Probes) over night at 4 °C and three further washing steps with PBS, embryos were mounted and analyzed by laser scanning confocal microscopy (SP5; Leica, Wetzlar, Germany). Staining of whole embryos for LacZ was as described previously^71^.

### Alizarin Red and Alcian Blue stainings

For skeletal staining, embryos were fixed in 95% ethanol for more than four days, after removal of fat and connective tissue, incubated in acetone for one day and stained with 0.15% alcian blue 8GS (Sigma-Aldrich)/0.05% alicarin red S (Sigma-Aldrich)/5% acetic acid in 70% ethanol at 37 °C for two days. After bleaching in 1% KOH for 12 to 48 hours embryos were destained with graded washes of glycerin (20% glycerin in 1% KOH, 50% glycerin in 1% KOH and 80% KOH in 1% glycerin). Embryos were stored in 100% glycerin.

### Histological analysis

Embryos and dissected organs from adult mice were fixed in 4% PFA in PBS, transferred to ethanol and embedded in paraffin. Longitudinally-sectioned paraffin-embedded embryos and organs were stained with hematoxylin and eosin. For CD 31 staining paraffin sections were stained as described previously^72^. Sections from dorsal chambers were de-paraffinized and rehydrated in xylene and isopropanol and antigens retrieved by boiling in Na3 citrate. Rabbit polyclonal anti-mouse Estrogen Receptor α antibody (Millipore) was used for detection and visualized by secondary goat anti-rabbit Alexa-Fluor 555 (Invitrogen) using microscopes and cameras from Zeiss and Olympus.

### Determination of *Rictor* mRNA expression levels in endothelial cells from thoracic aorta

*Rictor* deletion was induced in VECad-CreER^T2+^; *Rictor*^floxed/floxed^ mice at an age of four weeks with five consecutive intraperitoneal tamoxifen (Tx) injections according to the protocol of Monvoisin *et al.*^22^. (2 mg Tx/ml dissolved in corn oil at a concentration of 80 mg/kg bodyweight, T5648, Sigma-Aldrich). Littermate control mice were injected with corn oil alone. This injection protocol was used for all experiments in this study.

Aortae were excised from 6 month old Tx-injected (*Rictor*^iΔec^) and control mice (Ctrl), cleaned from adhering tissue and opened longitudinally. Endothelial cells were carefully scraped directly in RLT buffer (Qiagen) and RNA was extracted using the RNAeasy micro kit (Qiagen) according to the recommendations of the manufacturer. Equal amounts of RNA were transcribed to cDNA by WT (Whole Transcript)-Ovation™ Pico RNA Amplification System (NuGEN). Quantitative real-time PCR was performed using a SYBR green-based standard protocol. Primer list is shown in section ‘Real-time quantitative reverse transcription polymerase chain reaction’. Specificity of the primers was tested by melt curve and agarose gel analysis and sequencing. Relative expression levels were calculated using the comparative ΔCt method^73^.

### Isolation of endothelial cells

Mouse aortic endothelial cells (MAECs) were isolated from aortae of 8–10 weeks old *Rictor* floxed male mice as described earlier^74^,^75^. Briefly, fibrin gels were prepared by mixing 3 mg/ml of fibrinogen with serum-free DMEM complemented with non-essential amino acids, Sodium Pyruvate, Pen-Strep and thrombin on ice. 24-well plates were coated with the prepared fibrin gel allowed to polymerize at 37 °C. The excised aorta was cleaned, cut in small rings and placed on top of the gel and overlaid by fibrin gel. After gel had polymerized pre-warmed growth media (serum-free DMEM that contained 10% FCS, 200 μg/ml of ECGS, 10 ng/ml FGF2 and 0.1 IE/ml of heparin) was loaded to the wells. To protect the fibrin gel from degradation, 300 μg/ml of ε-amino caproic acid (Sigma A-7824) diluted in PBS was added to all wells every other day. After 10 days of culturing, capillary-like sprouts were observed under the microscope. The outgrown cells were harvested by pipetting up and down the fibrin gel. The gel-cell mixture was transferred to a six-well plate which had previously been coated with 0.1% gelatin gold, and 1 ml/well of growth media w/o heparin was added. The next day, cells were washed once with warm PBS and new EC growth media was added. Confluent cells were split 1:2 by trypsinization using TrypLETM-Express and characterized with endothelial cell specific immuno-fluorescent marker von Willebrand Factor (VWF, LabForce AG).

### Cell culture and treatments

For all experiments using MAECs, cell culture dishes were coated with 0.1% gelatin gold (Carl Roth GmbH 4274.1) for 20 minutes at 37 °C. MAECs were maintained in DMEM (Biochrom FG435), complemented with 10% or 1% (complete or starvation medium) FCS (Biochrom S0615), 1% sodium pyruvate (GIBCO 15140), 1% non-essential amino acids (GIBCO 11140) and 1% penicillin-streptomycin (GIBCO 15140). Stimulation with growth factors always included addition of heparin at a fixed ratio (1 IU heparin per 1.5 μg/ml FGF).

### Generation of *Rictor* ko cells

*Rictor* knockout was induced by adenoviral transfection of Cre-Recombinase on *Rictor* floxed MAEC. 2.5 × 10^5^ *Rictor* floxed MAECs were seeded on a 6 cm culture dish. The next day, the media was removed and a virus (100 MOI) that contained either Ade-CRE-GFP (Vector Biolabs 1045) or Ade-CMV-GFP (Vector Biolabs 1060) was added in 1 ml of growth media to the cells. After 7 hours, the media was removed, replaced by 4 ml of normal growth media, and incubated at 37 °C. Endothelial cells expressed GFP the following day. Down-regulation of *Rictor* and disruption of mTORC2 signaling was assessed by using qRT-PCR and western blotting 3 days after transfection (see also Fig. 4 lower left panel). Steps that involved handling viruses or virus-transfected cells were performed in a level-2 biosafety hood (Skan VSB 90) in a certified cell culture laboratory.

### Endothelial network-formation assay *in vitro*

Angiogenesis *in vitro* was assessed on the basis of a tube formation assay. Twenty-four-well culture plates (Costar; Corning) were coated with growth factor-reduced matrigel (BD Biosciences) in a total volume of 150 μL and allowed to solidify for 30 min at 37 °C. Tracking dye green (CytoPainter, abcam ab138891) was dissolved in 100 μl DMSO (=stock solution (1000×)). 20 μl of stock solution was mixed with 5 ml assay buffer. Cells were washed once with PBS, and 5 ml tracking dye green working solution added to the cells and incubated for 45 min at 37 °C in an CO_2_ incubator. Labeled MAEC were trypsinized and resuspended to a concentration of 10^5^/mL in DMEM/1% FCS. 500 μl of the cell suspension were added into each well and complemented with diluent (heparin) and growth factors (FGF2; VEGFA 25ng/ml with heparin). Then the cells were incubated at 37 °C for eighteen hours. The appearance of endothelial network was observed under an inverted microscope by fluorescence (FITC; Ex/Em = 490/520 nm) with a 4x objective (IX70, Olympus) and photographed. Number of master segments were quantified automatically using the Macro ‘Angiogenesis Analyzer’ by Gilles Carpentier (Gilles Carpentier. Contribution: Angiogenesis Analyzer, ImageJ News, 5 October 2012) for NIH Image J 1.47v Program.

### Immunoblotting

To extract protein, cells were washed twice with ice cold PBS and then harvested by scraping with a cell scraper (BD Falcon 353089) into 1 ml of ice cold PBS. The cell suspension was collected with a 1 ml pipette, transferred to a 2 ml Eppendorf tube, and then centrifuged for 5 minutes at 14,000 rpm at 4 °C. The pellet was resuspended in RIPA buffer (50 mM TrisHCl (pH7.4), 150 mM NaCl, 1 mM EDTA(pH8), 1% Triton X-100, 0.1% SDS, 0.25% Na-deoxycholate) that contained a complete mini protease inhibitor (Roche 11836153001) and a phosphatase inhibitor cocktail 2 (Sigma P5726). The sample was centrifuged for 15 minutes at 14,000 rpm at 4 °C. The resulting supernatant was transferred to a new 1.5-ml Eppendorf tube and the protein concentration measured by using a BCA protein assay kit (Thermo scientific 23223). Equal amounts of protein were loaded onto a 8% acrylamide-SDS gel. Separated proteins were transferred to a nitrocellulose membrane (Whatman BA85) using a semidry blotting procedure, blocked for 1 hour at room temperature (RT) in 5% Bovine Serum Albumine (BSA, Sigma-Aldrich A7906) in Tris buffered saline complemented with 0.1% Tween (TBS-T). Membranes were washed once with TBS-T and incubated overnight at 4 °C with one of the following primary antibodies against *Rictor* (#2140), Phospho-AKT (Ser^473^) (#9271), Phospho-AKT(Thr^308^) (#9275), AKT(#9272), PKCα, Phospho-PKCα (Ser^657^)(Santa Cruz Biotechnology, #12356), Phospho-ERK1/2 (Thr^202^/Tyr^204^) (#9101), ERK1/2 (#9102), Phospho-S6K1 (Thr^389^) (#9205), S6K1 (#9202) and ) and Phospho-S6 RP (Ser^235/236^) (#4858), all from Cell signaling (BioConcept, Switzerland) in a dilution of usually 1:1000. Mouse anti β-Actin 1:10,000 (Sigma A-5441) was used to control equal loading. The next day, the membrane was washed three times in TBS-0.1% tween and incubated with a secondary antibody goat anti rabbit HRP 1:5,000 (Cell Signaling 7074) or anti mouse HRP 1:50,000 (Cell Signaling 7076) for 1 hour at room temperature (RT). After another wash step, a chemiluminescent substrate that is used for the detection of HRP (Thermo Scientific 34080) was applied to the membrane, and then the membrane was incubated for 1 minute. The signal was detected with a CL-XPosure film (Thermo Scientific 34088), and the resulting bands were quantified by using ImageJ (Wayne Rasband, NIH, MD, USA).

### Real-time quantitative reverse transcription polymerase chain reaction

qRT-PCR was performed as described previously^76^,^77^. RNA was isolated using the RNAeasy Mini kit (Qiagen, Hilden, Germany), followed by an on column DNA digestion (Qiagen, Hilden, Germany). cDNA was transcribed from total RNA using Omniscript RT kit (Qiagen, Hilden, Germany) and random primers (Roche, Basel, Switzerland). To control for DNA contamination in the qRT-PCR, for each sample a control reaction missing reverse transcriptase was additionally amplified. Primers used are listed (5′ → 3′) below (Table 1). Primers were tested by cDNA dilution series to obtain optimal reaction conditions. qRT-PCR was performed using an iCycler iQ Real Time PCR Detection System (Biorad, Reinach, Switzerland) and iQ™ SYBR® Green Supermix (Biorad). Melting curve of each representative reaction was analyzed. The qRT-PCR was quantified using the formula: 2^−ΔC^_T_ = C_T_ gene of interest – C_T_ Tubulin^73^.

### Proliferation assay

4500 cells with 100 μl of growth media were seeded in a 96-well plate in 10 replicates. After serum-starvation (0.5% FCS) for 26 hours, cells were stimulated with 5–25 ng/ml of FGF2 or VEGFA, 10% FCS or 100μg/ml of insulin. After 72 hours, 10 μl of WST-1 solution (Roche Molecular Diagnostics) was added for 2 hours. Absorption was measured (A_450nm_-A_690nm_) by using a Spectramax M2 reader (Molecular Devices).

### Migration assay (wound healing)

MAECs were grown to confluency, starved (0.5% FCS) for 24 hours. A straight scratch using a 200 μl sterile tip was applied to the confluent monolayer (Star Lab, #S1120-8810). The monolayer was stimulated with diluent and FGF2 (25 ng/ml). The migration of the cells was photographed with an inverted microscope (Olympus IX71) at different time points (0, 1, 3, 6 and 9 hours after the stimulation) and measured with the software T-Scratch^®^ (Tobias Gebäck and Martin Schulz, ETH Zürich, 2008).

### Mouse dorsal skin fold chamber and matrigel sealing

To study the capillary remodeling and angiogenesis *in vivo* we used the dorsal skin fold chamber as described previously^33^ with a novel modification in order to deliver growth factors via matrigel to the tissue. For chamber implantation, two symmetrical titanium frames were mounted on the dorsal skin fold of the animal. One skin layer and the underlying fat were then completely removed in a circular area of 15 mm in diameter, and the remaining layers (consisting of striated skin muscle, subcutaneous tissue and skin) were covered with NaCl_2_ 0.9% and a glass cover slip incorporated into one of the titanium frames^33^. The animals were allowed to recover for two days. Skin was detached from the underlying muscle and removed in a circular area of 7 mm in diameter from the back of the chamber. Growth-factor reduced matrigel was mixed with FGF2 (1.5 μg/ml) and Heparin (5 IU units) or Heparin alone. The defect on the back of the chamber was sealed with 16μl of the matrigel mixtures, allowed to polymerize, and covered with a glass cover slip.

### Intravital microscopy

Repetitive intravital microscopic analyses of skin microvasculature were carried out daily over a time period of 7 days. Microscopic images were taken at 8 different areas within the center and the periphery of the wound. After injection of 0.2 ml FITC-labeled dextran (2%; MW 70000, Sigma-Aldrich, Munich, Germany) the microcirculation was visualized by intravital fluorescence microscopy. (Leica DM/LM; Leica Microsystems, Wetzlar, Germany). Microscopic images were captured by a CCD television camera (Kappa Messtechnik, Gleichen, Germany) and recorded on video (50Hz; Panasonic AG-7350-SVHS, Tokyo, Japan) for subsequent off-line analysis. Using ×10 (N-Plan ×10/0.25 LD, Leica), ×20 (HCX Apo ×20/0.50W, Leica) objectives blood flow was monitored in capillaries of the superficial and deep dermal plexus of the skin muscle. The epi-illumination setup included a mercury lamp with a blue filter (450–490 nm/>520 nm excitation/emission wavelength) and a green filter (530–560 nm/>580 nm).

### Matrigel plug assay

The matrigel plug assay was performed as previously described^78^. In brief, 8-week-old C57BL/6 mice were injected subcutaneously with 0.2 ml of matrigel containing 1.5 μg/ml bFGF with 1 μl (5 IU) Heparin. The injected matrigel rapidly formed a single, solid gel plug. After 7 days, mice were euthanized, the skin was pulled back to expose the matrigel plug. The matrigel plug was removed, photographed and fixed in formalin and paraffin embedded. Hemoglobin content was estimated by measuring mean pixel densities from five random 200 × 200 pixel areas from the magenta channel in CMKY converted images from each plug by the NIH ImageJ program.

The Matrigel plugs were removed and fixed in 4% buffered formalin. After 48 h fixation, the plugs were trimmed, dehydrated in graded alcohol and routinely paraffin wax embedded. Sections (3–5 μm thick) were prepared, mounted on glass slides, de-paraffinized in xylene, rehydrated through graded alcohols and stained with hematoxylin and eosin (HE) for the histological examination. For analysis of immunestainings, sections of one plug were photographed in three different areas (350 × 350 pixels), quantified and averaged. The n number refers to the mean of 3 areas of one plug. E.g. for microvessel invasion n = 4/7 refers to 4 groups of 3 averaged areas from control plugs compared to 7 groups of 3 averaged areas from 7 *Rictor* ko plugs. Immunohistology for CD31 antigen was employed to highlight endothelial cells. Anti-CD-31 immunohistochemical staining was performed according to manufacturer’s protocol (Lifespan Biosciences). Slides were photographed using a brightfield microscope (Zeiss Axioskop 2, Germany). Immunohistology for the CD68 antigen was employed to highlight cells of the monocyte/macrophage lineage^79^. Briefly, sections were deparaffinized in xylene (2 × 5 min) and rehydrated in decreasing concentrations of ethanol (2 × 3 min washes in 100% ethanol, followed by 1 × 3 min wash in 96% ethanol). Sections were washed twice with Tris-buffered saline (TBS, 0.1 M Tris–HCl with 0.9% NaCl, pH 7.4) and incubated for 1 h at 37 °C with the primary antisera (1:100, ab125212, Clone KP1, Abcam, Cambridge, United Kingdom), after heat pre-treatment in citrate acid (0.01M, pH 9.0) in a 97 °C water bath for 20 min. An anti-rabbit IgG DAB detection system was subsequently applied according to the manufacturer’s protocols (Discovery OmniMap anti-Rb HRP, Roche, Basel, Switzerland). Sections were then washed 3 × in TBS and 1 × in distilled water and counterstained for 1 min with hematoxylin, followed by rinsing for 5 min in tap water and dehydration in ascending alcohols, clearing in xylene, coverslipping and mounting. Sections of murine immune system organs were used as positive control. All slides were scanned using digital slide scanner NanoZoomer-XR C12000 (Hamamatsu, Japan) and images were taken using NDP.view2 viewing software (Hamamatsu).

### Statistical analysis

Statistical tests were performed by GraphPad Prism 5.04 software (San Diego, CA, USA). On a general basis, two-way analysis of variance (ANOVA), followed by a Bonferroni-post test comparing all pairings was calculated whereby a P value of less than 0.05 was considered as statistically significant. For the comparison of two groups, the unpaired t-test was used. Datapoints in graphs represent average values ± standard error of the mean unless otherwise stated.

## Additional Information

**How to cite this article**: Aimi, F. *et al.* Endothelial *Rictor* is crucial for midgestational development and sustained and extensive FGF2-induced neovascularization in the adult. *Sci. Rep.* **5**, 17705; doi: 10.1038/srep17705 (2015).

## Supplementary Material

Supplementary video 1

Supplementary video 2

Supplementary video 3

Supplementary video 4

Supplementary Information

## Figures and Tables

**Table 1:** 

Gene	Forward	Reverse
*Rictor*	TGC GAT ATT GGC CAT AGT GA	ACC CGG CTG CTC TTA CTT CT,
*Gapdh*	AAA TGG TGA AGG TCG GTG TG	GTT GAA TTT GCC GTG AGT GG,
*Tubulin (Tuba1a)*	TCA CTG TGC CTG AAC TTA CC	GGA ACA TAG CCG TAA ACT GC
*β-actin (Acta1)*	CGT GCG TGA CAT CAA AGA GA	CCC AAG AAG GAA GGC TGG A
*mFlt1*	GCT TCT GGA GGA CGT CAA CA	TCC CGA TAC AGG GCT TCA GA
*sFlt1*	CTC CTC TGT CCA CCC AGG TA	CTG CAC TTT TGC CGT CAG TC
*VegfA*	TTC GTC CAA CTT CTG GGC TC	CGA GCT AGC ACT TCT CCC AG
*KDR*	GGA AGG CCC ATT GAG TCC AA	GTT GGT GAG GAT GAC CGT GT
*FgfR1*	CGT AGG CCT GTA GCT CCC TA	TGA ACT TCA CCG TCT TGG CA
*Pkcα*	GGA ATG AGT CCT TCA CGT TCA AA	TTA GCT CTG AGA CAC CAA AGG
*MCP1 (Ccl2)*	CAG GTC CCT GTC ATG CTT CT	GTG GGG CGT TAA CTG CAT CT
*Vcam1*	GGC TGC GAG TCA CCA TTG	GCA CAG GTA AGA GTG TTC ATT C
*Icam1*	GAC GCA GAG GAC CTT AAC AG	GAC GCC GCT CAG AAG AAC
