# Supplementary material for: Endothelial Rictor is crucial for midgestational development and sustained and extensive FGF2-induced neovascularization in the adult
Source: Sci Rep. 2015 Dec 4;5:17705. doi: 10.1038/srep17705 (PMC4669526; doi:10.1038/srep17705)
Supplement: Supplementary Information [file srep17705-s5.pdf]

Endothelial *Rictor* is crucial for midgestational development and sustained and extensive FGF2-induced neovascularization in the adult

Fabio Aimi[1+], Stavroula Georgiopoulou[1+], Ina Kalus[1], Fabienne Lehner[1], Alica Hegglin[2], Përparim Limani[4], Vinicius Gomez de Lima[1], Markus Rüegg[3], Michael N. Hall[3], Nicole Lindenblatt[2,5], Elvira Haas[1], Edouard J. Battegay[1,5,6], Rok Humar[1,5 *]

[1] Department of Internal Medicine, University Hospital, CH-8091 Zürich, Switzerland

[2] Division of Plastic and Reconstructive Surgery, University Hospital, CH-8091 Zürich, Switzerland

[3] Biozentrum, University of Basel, CH-4057 Basel, Switzerland

[4] Division of Visceral and Transplant Surgery, University Hospital, CH-8091 Zürich, Switzerland

[5] Zürich Center for Integrative Human Physiology, University of Zürich, Switzerland

[6] Center of Competence Multimorbidity and University Research Priority Program "Dynamics of Healthy Aging", University of Zurich, Switzerland

[+]These authors contributed equally to this manuscript

*Please address correspondence to:

Dr. Rok Humar

University Hospital Zürich

Division of Internal Medicine

Wagistrasse 12

CH-8952 Zürich-Schlieren, Switzerland

Tel: +41 44 556 3235

Fax: +41 44 556 3232

Email: Rok.Humar@usz.ch

Keywords: Angiogenesis, FGF2, mTORC2, Rictor, VEGF

Short title: Endothelial mTORC2 controls aberrant angiogenesis

Supplementary Information

Suppl. Figure 1

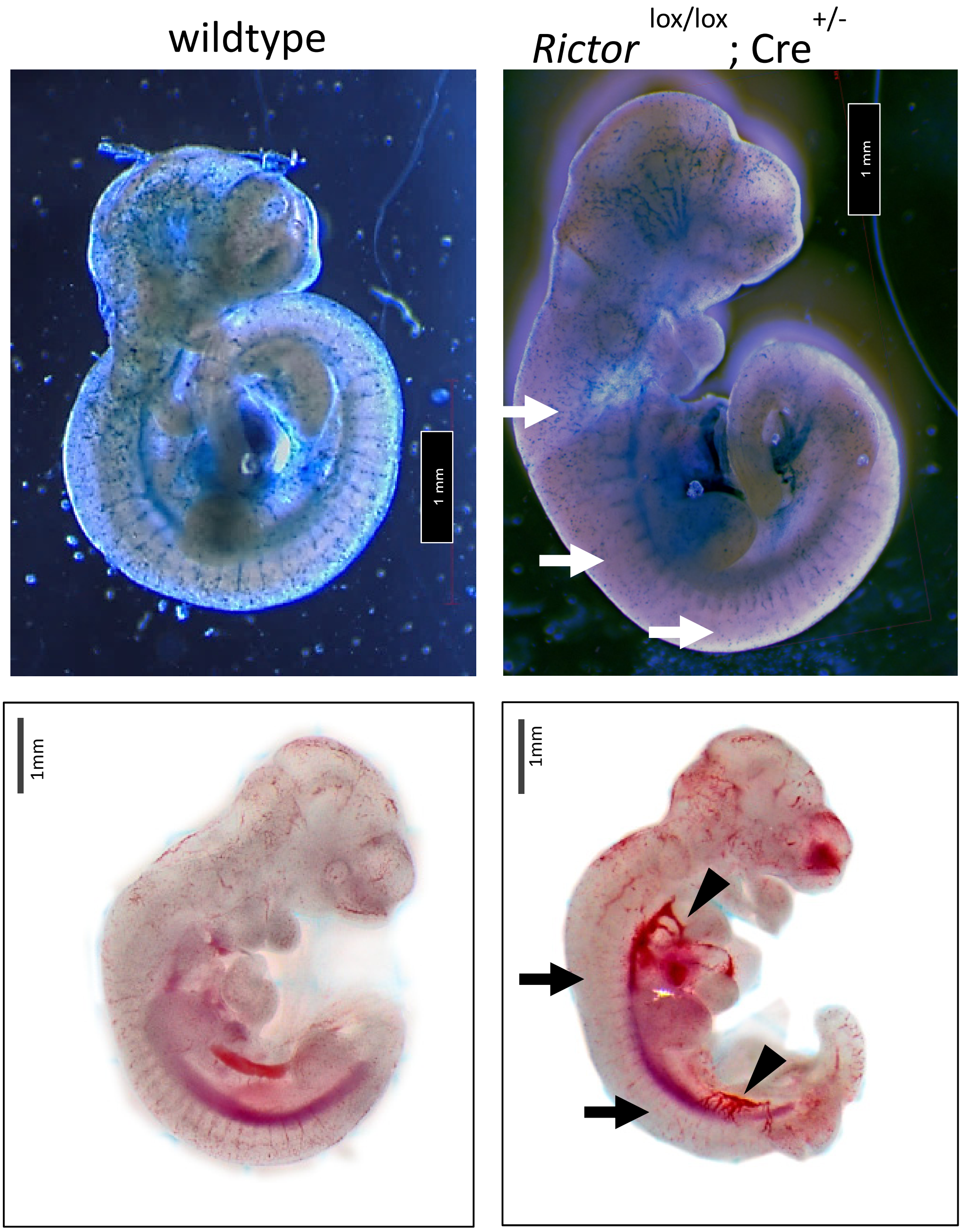

Fig S1. ***Rictor*** $^{\Delta ec}$ **embryos display reduced peripheral vascularization.** Representative β-galactosidase staining (blue) of E10.5 embryos (upper panels) shows the active sites of VE-Cadherin-Cre recombination. Images below show representative E10.5 embryos before staining. Arrowheads on the right indicate distinct vascular remodeling in *Rictor* knockout embryos (detected in 2 out of 11 *Rictor* knockout embryos). Arrows indicate reduced LacZ-positive staining and reduced peripheral vasculature in *Rictor* knockout embryos (detected in 7 out of 11 *Rictor* knockout embryos).

Suppl. Figure 2

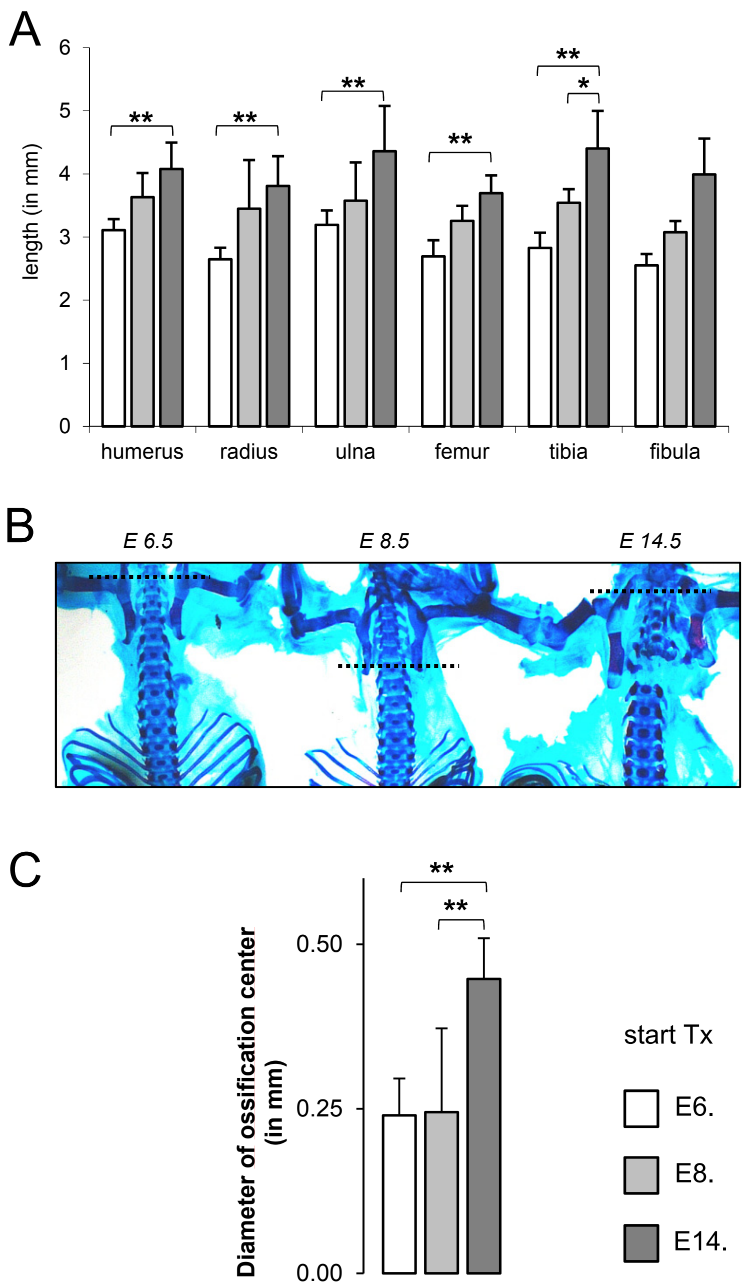

Fig S2. ***Rictor*** **$^{i\Delta ec}$ embryos display a delay in ossification. A.** Quantification of length of long bones of the upper limb (humerus, radius und ulna) and lower limb (femur, tibia and fibula) of endothelial specific *Rictor* deficient embryos injected with Tx at E6.5 (white bars), E8.5 (grey bars) and E14.5 (dark bars) as starting time point. N=4, Students t-test ** p <0 .01 * p < 0.05 compared to E14.5 **B.** Representative pictures of the lower spine of embryos stained with alizarin red (bone) and alcian blue (cartilage) for skeletal analysis indicated starting time points for Tx injections. Dotted line: border ossificated vertebrae. **C.** Statistical analysis of diameter of ossification centers in lumbal vertebrae upon knock out of *Rictor* at indicated starting time points compared to E14.5. N=4, Mann-Whitney Rank Sum Test ** p < 0.01

Suppl. Figure 3

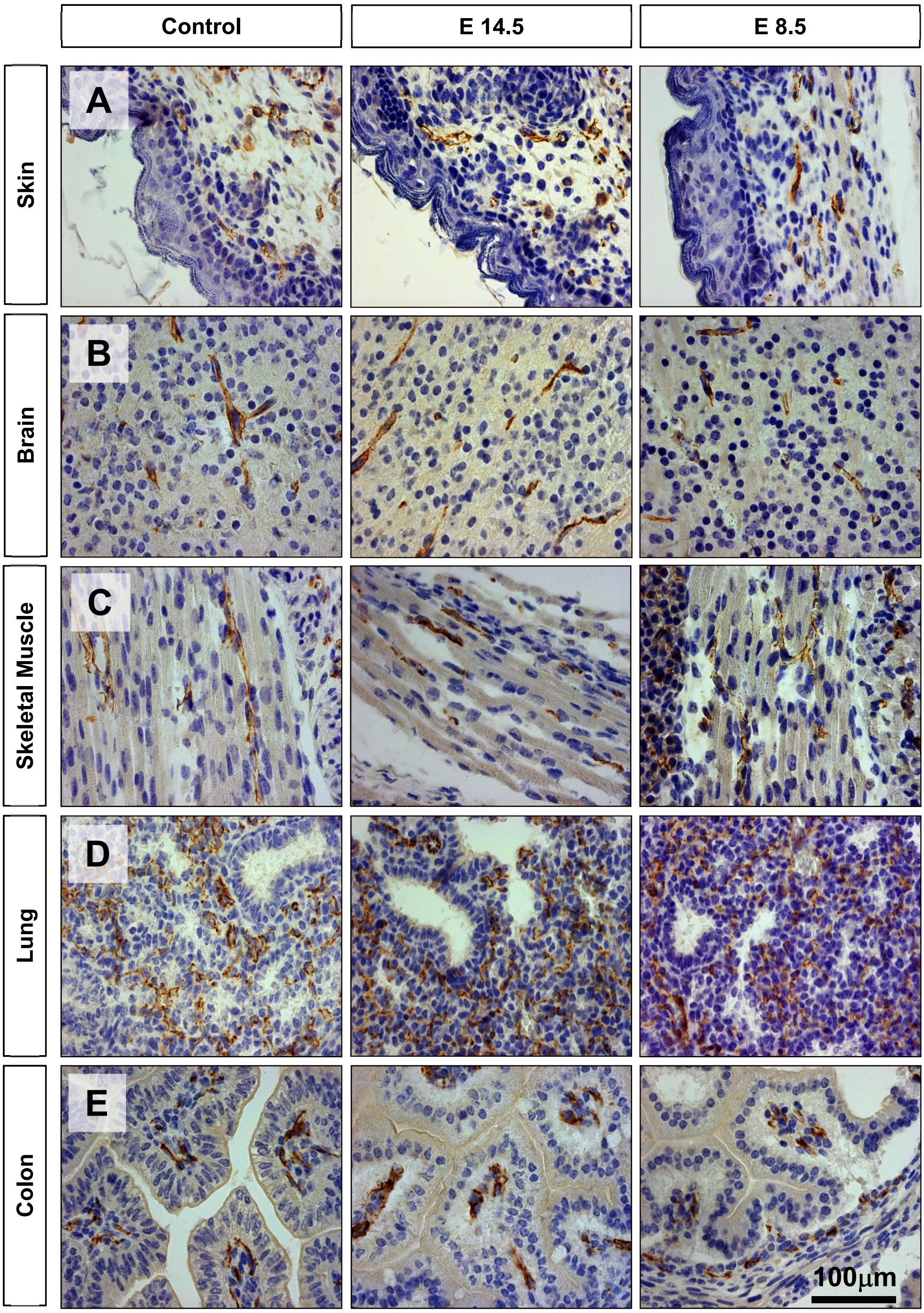

Fig S3. Histological analysis of *Rictor* $^{i\Delta ec}$ embryos with Tx-injections starting at E 8.5 and E14.5 in comparison to control embryos. Embryos were harvested at E17.5, fixed, embedded in paraffin and longitudinally sections were immunohistologically stained with anti CD31 antibody to detect endothelium. **A:** skin, **B:** brain, **C:** skeletal muscle, **D:** lung and **E:** colon. CD31, brown; nuclear counterstain, blue. Scale bar = 100 mm

Suppl. Figure 4

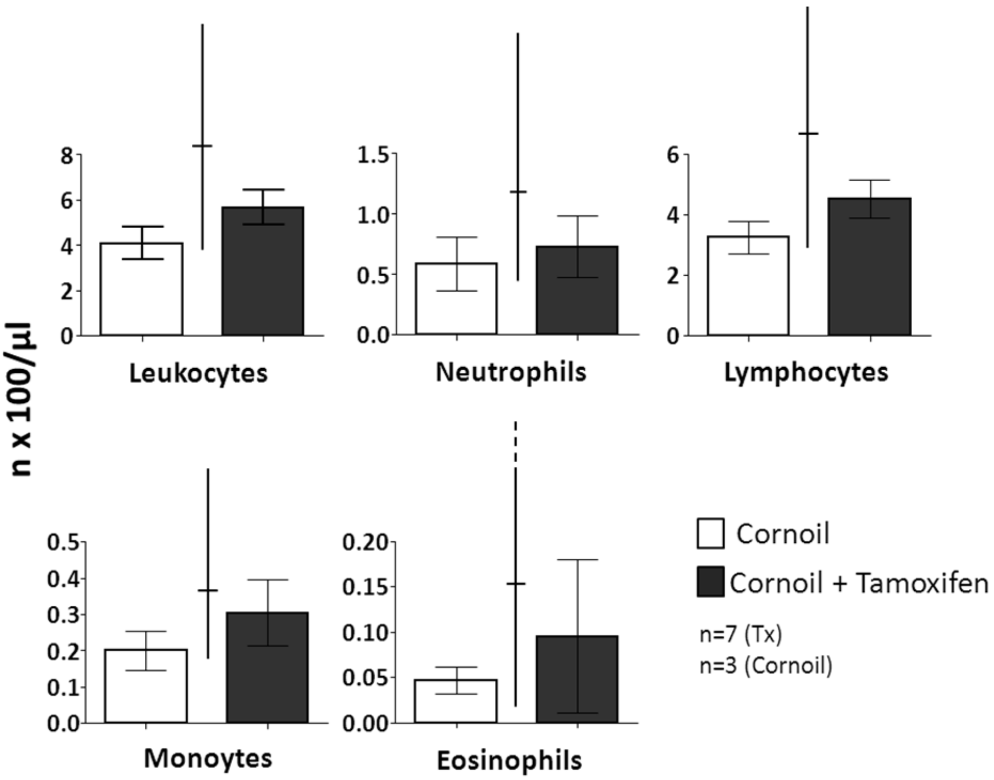

Fig S4. Endothelial *Rictor* knockout does not modulate hematological profile. Hematological profile (Count of leukocytes, neutrophils, lymphocytes, monocytes and eosinophiles) was assessed from 10 weeks old Cornoil and Tamoxifen/Cornoil-injected male $Cre^{+/+}$; *Rictor*$^{lox/lox}$ mice. Line between bars indicates normal range of parameters for C57/Bl6 mice.

Suppl. Figure 5

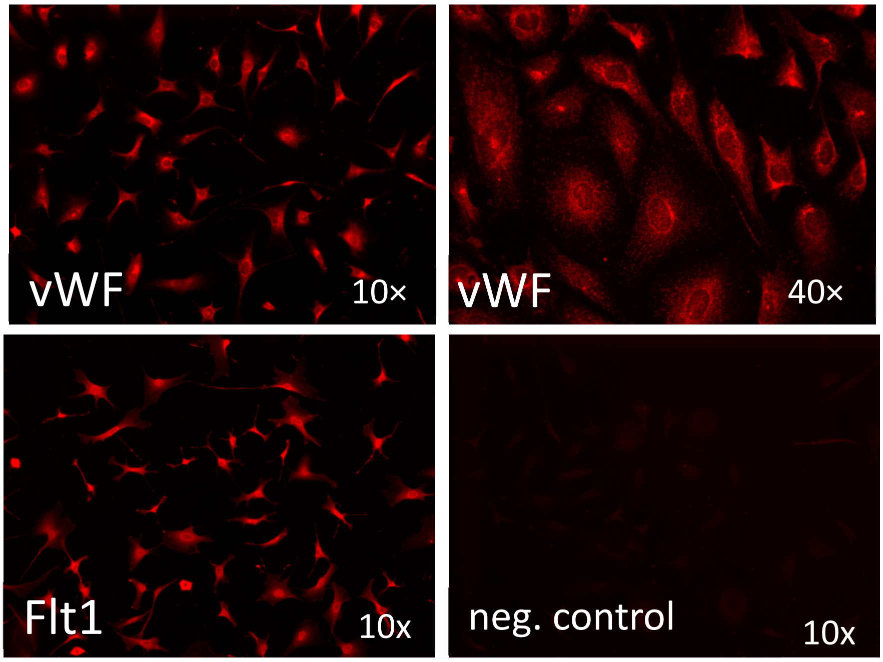

Fig S5. Characterization of endothelial cells. Fluorescent Immune-staining (red) of a representative endothelial cell isolate for endothelial cell markers von Willebrand Factor (vWF) and VEGF receptor 1 (Flt1). The 40x magnification of vWF-staining shows a vWF-typical granular pattern. As negative control, a staining without primary antibody is shown.

Suppl. Figure 6

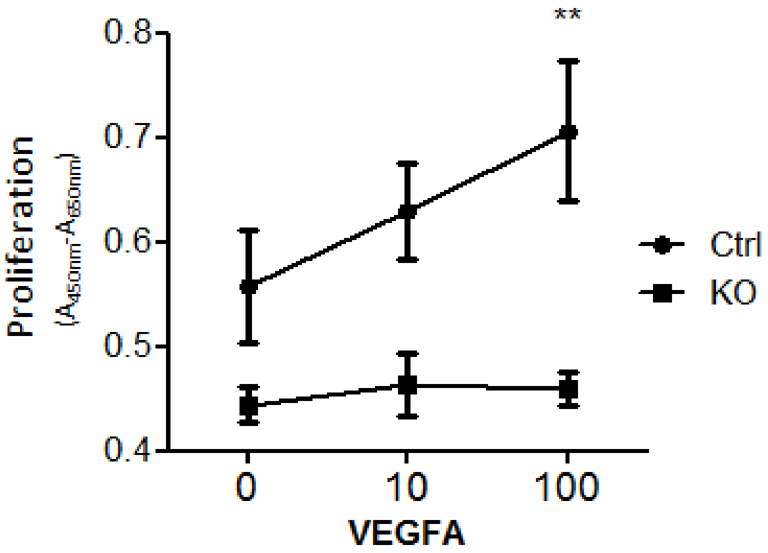

Fig S6. Endothelial *Rictor* ko decreases VEGFA-induced MAEC proliferation. Control and *Rictor* ko MAEC were seeded subconfluently, cultured for 25 hours in growth medium with 0.5% FCS and then stimulated with diluent, 10 ng/ml and 100 ng/ml of VEGFA. Cell proliferation was measured by WST-1 reagent. Points (±SE) represent absolute proliferation values (Absorption=A450nm-A650nm) in FGF2 stimulated wildtype (circles) or *Rictor* ko (squares) MAEC. Proliferation was significantly ($P<0.001$, $n_{exp}=3$) decreased in *Rictor* ko MAEC compared to controls at 100 ng/ml VEGFA stimulation.

Suppl. Figure 7

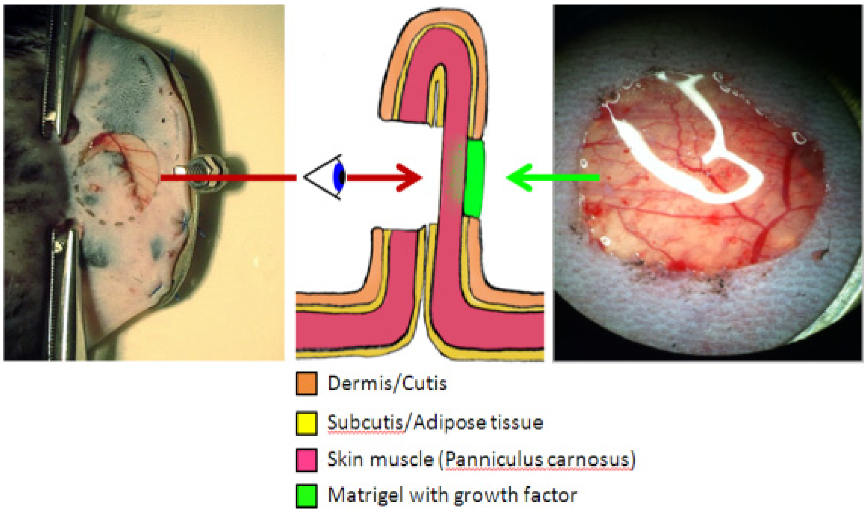

Fig S7. Modification of the dorsal skinfold chamber. Skin was detached from the underlying muscle and removed in a circular area of 7 mm in diameter from the side opposite to the observation window of the chamber. This defect on the back of the chamber was sealed with 20 µl growth factor-reduced matrigel containing heparin (5 IU) with or without FGF2 (1.5 µg/ml). Afterwards, it was covered with a glass cover slip incorporated into the titanium frame.

Suppl. Figure 8

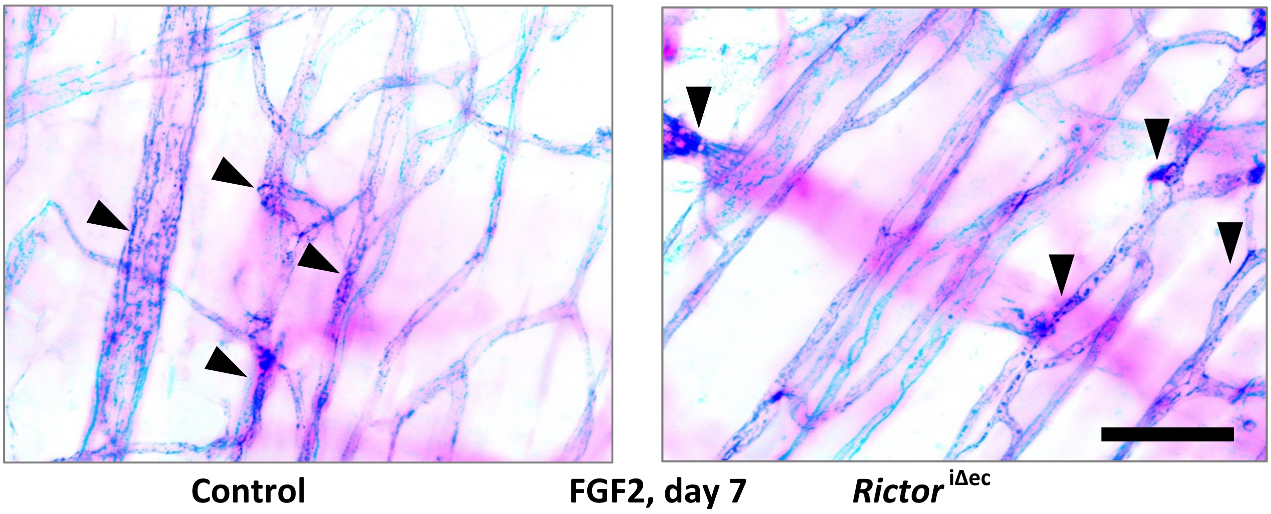

Fig S8. Fluorescent intravital staining for ricinus communis agglutinin I (RCA I). a galactose-binding lectin from castor beans, that binds to endothelial cells at sites of plasma leakage. Methods: 50 µl (1 µg/µl) of TRITC-RCA-I (Vector labs) in PBS was injected via tail vein in anesthetized mice carrying a dorsal skinfold chamber for 30 min. Then, mice were euthanized and skin muscle dissected from the skinfold chamber observation window, and mounted on coverslips. Fluorescence was recorded by optical-grid sectioning of ca. 10×3 µm sections (Zeiss, Apotome 2, 25x magnification). Inverted image of orthogonal projections of are shown below (blue=leakage points, arrowheads mark regions with increased positive staining). Pilot experiment (n=2).

Suppl. Fig. 9

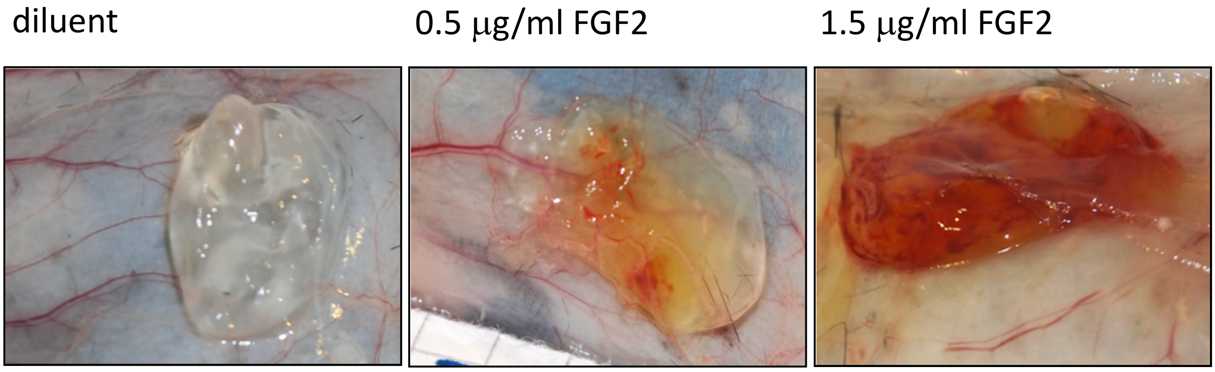

Fig S9. Dose response of FGF2-matrigel plugs in control mice. Diluent, 0.5 µg/ml and 1.5 µg/ml FGF2 containing matrigel plugs were implanted in each flank of each mouse. For experiments, plugs were removed 7 days post implantation and analyzed

Suppl. Fig. 10

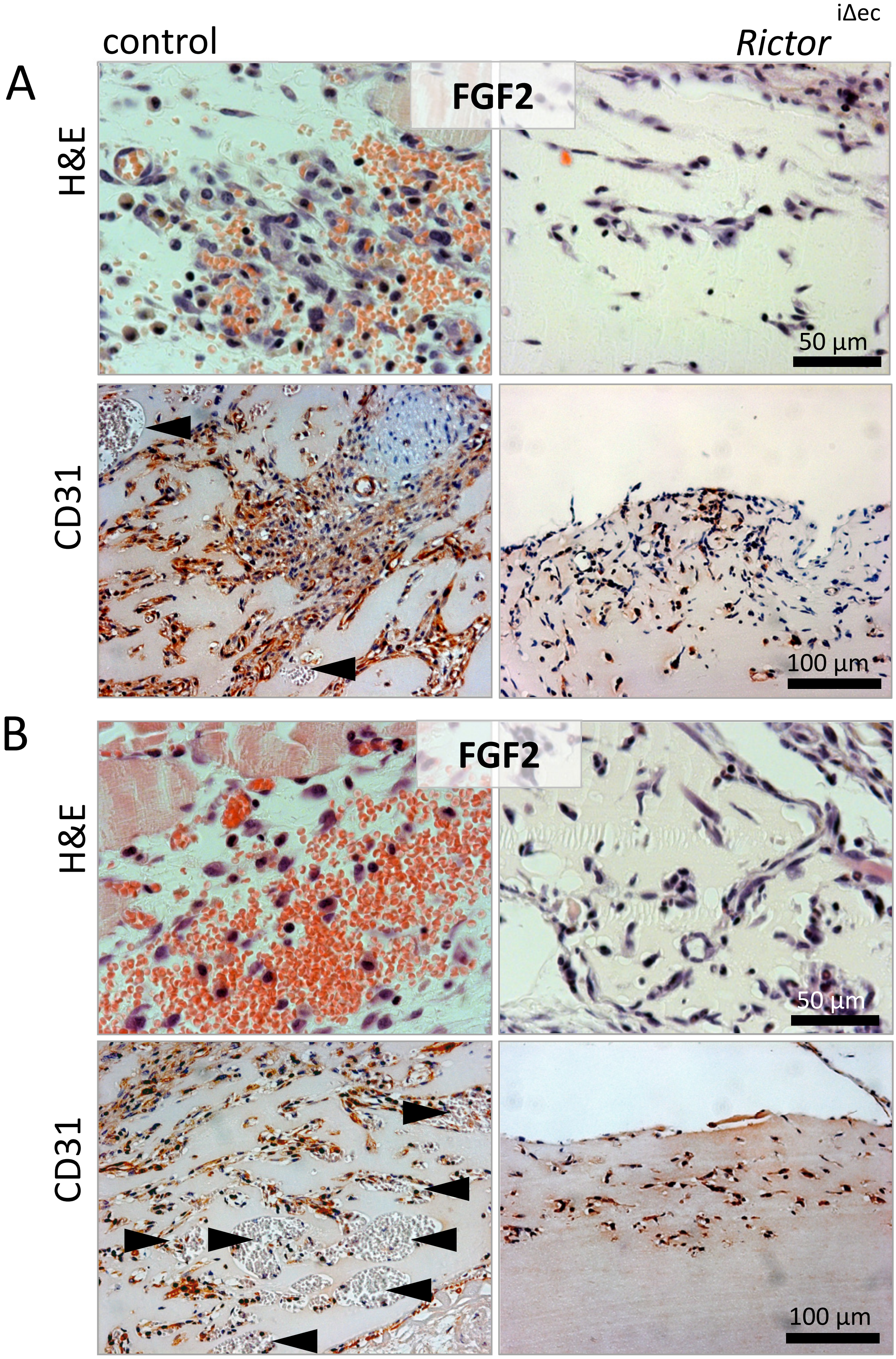

Fig S10. Hemorrhage in FGF2-matrigel plugs in control mice. Representative micrographs from 2 further sets of experiments (**A** and **B**) displaying hematoxylin and eosin stained (H&E) and CD31-stained matrigel areas showing local leakage and hemorrhagic areas in FGF2 containing plugs from control mice compared to plugs from *Rictor*$^{i\Delta ec}$ mice. Arrowheads point to local spots of leaked erythrocytes in FGF2-containg control plugs in CD31-stained matrigel areas.

Legends to supplementary videos

Supplementary Video 1

Representative intravital microscopic recording of unstimulated wound bed before matrigel sealing (baseline, 20× magnification) after injection of 0.15 ml 1% fluorescein isothiocyanate (FITC)-labeled 70 kDa dextran. First half of the video shows capillaries from control mice, second half shows capillaries from *Rictor*$^{i\Delta ec}$ mice.

Supplementary Video 2

Representative intravital microscopic recording of unstimulated wound bed 7 days after matrigel (diluent/heparin) sealing (day 7, 20× magnification). The microvasculature of the panniculus carnosus responded similarly to wounding and matrigel sealing in control and *Rictor*$^{i\Delta ec}$ mice.

Supplementary Video 3

Representative intravital microscopic recording of FGF2-stimulated wound bed 7 days after matrigel (FGF2/heparin) sealing (day 7, 20× magnification). Diameters measured on day 7 after FGF2 stimulation in *Rictor*$^{i\Delta ec}$ mice were significantly and homogeneously smaller compared to the control group on this day.

Supplementary Video 4

Representative intravital microscopic recording of FGF2-stimulated wound bed 7 days after matrigel (FGF2/heparin) sealing (day 7, 10× magnification). In control mice, tortuous and bulbous vascular structures with larger luminal diameters observed in capillaries and small draining arterioles and venules developed in control mice. In *Rictor*$^{i\Delta ec}$ mice, a restrained and different mode of remodeling emerged with thin-connecting anastomoses between capillaries and draining arterioles. Orientation of capillaries remained largely parallel in *Rictor*$^{i\Delta ec}$ mice after 7 days of FGF2 stimulation.
